# Enhancing near-infrared II photodynamic therapy with nitric oxide for eradicating multidrug-resistant biofilms in deep tissues

**DOI:** 10.1016/j.bioactmat.2023.11.006

**Published:** 2023-11-25

**Authors:** Fanqiang Bu, Xiaoxu Kang, Dongsheng Tang, Fang Liu, Lin Chen, Pengfei Zhang, Wenli Feng, Yingjie Yu, Guofeng Li, Haihua Xiao, Xing Wang

**Affiliations:** a State Key Laboratory of Organic-Inorganic Composites, Beijing Laboratory of Biomedical Materials, Beijing University of Chemical Technology, Beijing, 100029, PR China; b Beijing National Laboratory for Molecular Sciences, Key Laboratory of Polymer Physics and Chemistry and CAS Key Laboratories of Organic Solids, Institute of Chemistry, Chinese Academy of Sciences, Beijing, 100190, PR China; c Department of Oncology of Integrative Chinese and Western Medicine, China-Japan Friendship Hospital, Beijing, 100029, PR China; d College of Chemistry and Chemical Engineering, Qiqihar University, Qiqihar, 161006, PR China

**Keywords:** Photodynamic therapy, Near-infrared II region, Nitric oxide, Multidrug-resistant *Staphylococcus aureus*, In vivo biofilm model

## Abstract

Nitric oxide (NO) enhanced photodynamic therapy (PDT) is a promising approach to overcome drug tolerance and resistance to biofilm but is limited by its short excitation wavelengths and low yield of reactive oxygen species (ROS). Herein, we develop a compelling degradable polymer-based near-infrared II (NIR-II, 1000–1700 nm) photosensitizer (PNIR-II), which can maintain 50 % PDT efficacy even under a 2.6 cm tissue barrier. Remarkably, PNIR-II is synthesized by alternately connecting the electron donor thiophene to the electron acceptors diketopyrrolopyrrole (DPP) and boron dipyrromethene (BODIPY), where the intramolecular charge transfer properties can be tuned to increase the intersystem crossover rate and decrease the internal conversion rate, thereby stabilizing the NIR-II photodynamic rather than photothermal effect. For exerting a combination therapy to eradicate multidrug-resistant biofilms, PNIR-II is further assembled into nanoparticles (NPs) with a synthetic glutathione-triggered NO donor polymer. Under 1064 nm laser radiation, NPs precisely release ROS and NO that triggered by over-expressed GSH in the biofilm microenvironment, thereby forming more bactericidal reactive nitrogen species (RNS) in vitro and *in vivo* in the mice model that orderly destroy biofilm of multidrug-resistant *Staphylococcus aureus* cultures from clinical patients. It thus provides a new outlook for destroy the biofilm of deep tissues.

## Highlights

• The unique –(–D–A_1_–D–A_2_–)_n_– structure of PNIR-II is built.
• PNIR-II exhibits PDT efficacy without PTT under 1064 nm laser radiation.
• Under centimetre-level tissue barriers, PNIR-II can still maintain efficient ROS yield *in vitro.*
• The effectiveness of the NPs+1064 nm group in producing ROS, NO, and RNS is validated *in vivo.*
• NPs+1064 nm group can effectively remove biofilm in clinieal MDRSA-infected mice model under 2.2 cm tissue barriers.

## Graphical abstract

A photosensitive polymer (PNIR-II) excited by a 1064 nm laser, thus maintaining the 50 % efficiency of photodynamic therapy (PDT) at 2.6 cm tissue depth, is designed. Nanoparticles assembled with PNIR-II and the gas donor polymer PSNO exhibit good bactericidal and anti-biofilm properties *in vivo* biofilm model with 2.2 cm tissue barriers, demonstrating the great potential of NIR-II PDT.

**Figure:**
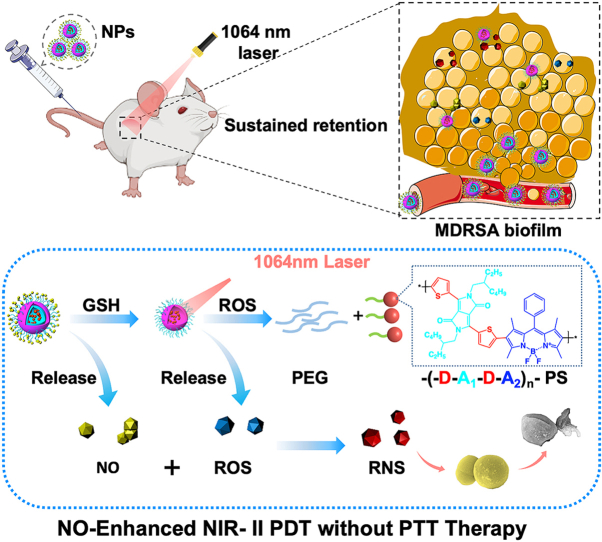

## 1 Introduction

Biofilm infection has been a tricky issue in clinics, especially the biofilm caused by multidrug-resistant (MDR) bacteria [1,2]. Photodynamic therapy (PDT) is a feasible anti-infection strategy because of its low invasiveness, high controllability and low toxicity [3,4]. However, PDT is inhibited by biofilms because (i) hypoxia levels in biofilms inhibit the production of reactive oxygen species (ROS) by photosensitizers (PSs) [5,6]; (ii) over-expression of glutathione (GSH) will directly consume ROS produced by PSs [7]; (iii) extracellular polysaccharides (EPS) prevent PSs from penetrating into biofilm [8,9]. For these reasons, combination therapies are usually used to enhance PDT. The combination therapies have the benefits of targeting different pathogenesis, synergistic or complementary treatment [[10], [11], [12], [13], [14]]. Specially, when combined with a nitric oxide (NO) precursor, PSs successfully break through biofilm barriers (including EPS) and counteract severe hypoxia [[15], [16], [17]]. Notably, NO continuously depletes GSH, destroys the biofilm and triggers immune responses. It can also interact with reactive oxygen species (ROS) to produce reactive nitrogen species (RNS, peroxynitrite anion) which displays better bactericidal effect [18,19]. Therefore, the combination therapy of NO-enhanced PDT is gradually being used to treat biofilm infections.

However, there are two reasons that limit the applications of PDT in the treatment of biofilm infections. First, excitation wavelengths of traditional PSs usually at the visible or near-infrared I (below 900 nm) spectra, which lead to reduced tissue penetration depth [20,21]. Among them, PSs excited by near-infrared I light penetrate depth about 3–6 mm underneath the skin, which is mainly used to treat epidermal (100 μm) and dermal (1–4 mm, vascular layer) infections [[22], [23], [24]]. Therefore, traditional PSs still face great challenges in eliminating infections above centimetre-scale tissue barriers. The newly emerging NIR-II (1000–1700 nm) phototherapy agents are expected to overcome the above problems [25,26]. It is important to develop new NIR-II PSs to address this issue, since the study of organic NIR-II PSs is still in its infancy.

Secondly, ensuring ROS yield is another challenge for anti-biofilm infection, and it is also a challenge for the design of the NIR-II PSs. The NIR-II molecules have a smaller S_1_→S_0_ energy gap (*ΔE*), the smaller *ΔE* increases the internal conversion (IC) rate [27]. Increasing the non-radiative IC process can improve the photothermal conversion efficiency (PCE) of NIR-II molecules, reducing radiative transition (RT) and intersystem crossing (ISC) [28,29]. This ultimately reduces the ROS yield and fluorescence quantum yield of NIR-II molecules. To date, the strategies to control the absorption wavelength and reduce the S_1_-T_1_ energy gap (*ΔE*_*S-T*_) of PSs by increasing the π-conjugated system of the donor-acceptor (D-A) structure have been successively proposed [30,31]. Tian et al. developed an A-D-A electronic structure that exhibits good PDT performance at 880 nm [32]. It is proved that the dual-acceptor structure is more conducive to the design of PSs [[33], [34], [35]]. Moreover, constructing a highly rigid D-A structures can effectively allocate more excitation energy to ISC transitions for PDT [[36], [37], [38], [39], [40]]. Based on these pioneering works, novel organic NIR-II PSs are expected.

**Scheme 1:**
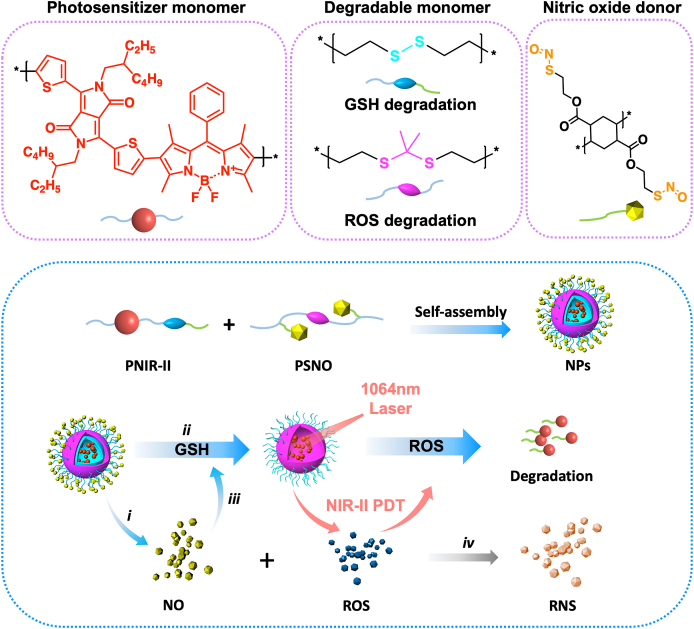
NIR-II laser-irradiated NPs eliminate MDRSA biofilm via NO-enhanced centimetre-level PDT. Cascade reaction of NPs prepared by mixing oxidation-sensitive PNIR-II with reduction-sensitive PSNO irradiated by a 1064 nm laser. (*i*) GSH-stimulated NPs release NO; (*ii*) disulphide bonds in PNIR-II decrease GSH; (*iii*) NO oxidises GSH to glutathione oxide (GSSG); (*iv*) NO reacts with ROS to produce RNS, which has greater killing efficiency than that of ROS and NO.

In this study, we developed versatile nanoparticles (NPs) by assembling polymeric NIR-II PS (donated as PNIR-II) with NO-donor polymer (denoted as PSNO) to overcome drug resistance and tolerance of bacterial biofilms (Scheme 1). First, PNIR-II with large conjugation and GSH-responsive structure was designed and prepared. A primary D–A_1_ conjugation was constructed from DPP and thiophene, and then BODIPY was introduced to form the –(–D–A_1_–D–A_2_–)_n_– cyclic conjugated structure. To improve the degradability and biocompatibility of PNIR-II, a GSH reduction-sensitive disulfide structure was introduced into the polymer chain. Thus, the obtained PNIR-II could be rapidly degraded by GSH. Meanwhile, the GSH-triggered NO-donor partner PSNO was developed to boost PDT efficiency. The nitroso-thiols were responsible for GSH-triggered release of NO. Besides, the oxidation-sensitive thioketal structures would degrade rapidly when exposed to ROS. As a result, GSH, NO and ROS formed a linkage. The multidrug-resistant *Staphylococcus aureus* (*S. aureus*, MDRSA) was collected from clinical patients, and then the mice MDRSA infection model was used to test the therapeutic efficacy of the NPs under NIR-II laser radiation. Scheme 1 depicts the potential mechanism of NPs-mediated biofilm eradication. Herein, biofilm infection site could exhibit an enhanced permeability and retention (EPR), similar to that of tumors. Because the inflammation induced by biofilm changes the vascular permeability, which leads to passive targeting of nanoparticles to biofilm infection site [41]. Owing to their small size, NPs were easily incorporated into the biofilms. Subsequently, these NPs released NO upon encountering the overexpressed GSH (i) and decreased GSH levels in a cascade reaction (ii). Simultaneously, the desulphated bonds further reduced GSH levels (iii) and induce self-degradation of PNIR-II. Moreover, exposure to the NIR-II laser light promoted the interaction of exciting ROS with NO to generate highly toxic peroxynitrite anion (•ONOO^−^); this could enhance PDT efficiency and trigger the active destruction of PSNO (iv). Furthermore, NPs+1064nmlaser still maintains effectiveness in removing biofilms under a 2 cm tissue barrier. Therefore, NO-enhanced NIR-II PDT is a promising approach to overcome drug resistance and tolerance to biofilms.

## 2 Materials and methods

### 2.1 Materials

Poly (ethylene glycol) (PEG, *M_w_* = 5000, 98 %), 2-hydroxyethyl disulfide, glutathione, 1,2,4,5-cyclo hexanetetracarboxylic dianhydride, polyethylene glycol-5K (PEG_5K_–OH), hyaluronidase, 1,2-Benzenedicarboxaldehyde (OPA) and 2,2,6,6-Tetramethyl-1-piperidinyloxy (TEMP, Spin trapping reagent) were purchased from energy chemical (Beijing, China). 3-(4,5-dimethylthiazol-2-yl)-2,5-diphenyltetrazolium bromide (MTT), tryptone soybean broth (TSB) and tryptic soy agar (TSA) were purchased from Solarbio (Beijing, China). Methanol, toluene, acetonitrile, glutaraldehyde, tetrahydrofuran, ethanol was purchased from Concord (Tianjin, China). Viability/Cytotoxicity Assay for Bacteria Live & Dead Cells kit, ATPase and BCA kit was purchased from Solarbio (Beijing, China). TNF-α Elisa kits (1 mg/mL), IL-6 antibody Elisa kits (1 mg/mL) and IL-10 antibody Elisa kits (1 mg/mL) were purchased from Beijing Boosen Biotechnology Co., Ltd (Beijing, China). DAF-FM DA, DCFH-DA and R21, were purchased from BestBio (Shanghai, China).

### 2.2 Preparation and characterization of polymer PNIR-II and PSNO

Supporting information demonstrated synthesis method and data.

### 2.3 Characterization

Dynamic Light Scattering (DLS) was performed by Malvern Zetasizer NanoZS90. The Transmission Electron Microscopy (TEM) were accomplished by using HT7700 (Hitachi) electron microscope. The morphology of bacteria was obtained by Scanning Electron Microscope (SEM) carried out with a JEOL JSM-7800F electron microscope. Molecular weight of polymer was characterized by Gel Permeation Chromatography (GPC) (GPC Waters 1515, Waters, USA). All OD values were measured by SpectraMax M3. Confocal Laser Scanning Microscopy (CLSM) was performed with Leica SP8. ^1^H NMR spectra were measured by a 400 MHz spectrometer (Bruker) at room temperature. UV–vis spectra were tested by an UV–vis spectrometer (U-4100, Hitachi, Japan). Fluorescence spectra were tested by a fluor spectrophotometer (F-7000, Hitachi, Japan). The electron paramagnetic resonance (EPR) spectra were tested by an electron paramagnetic resonance (EMX-500 10/12, Bruckner, Germany). Mice were imaged by Series-II 900/1700 (Shtips, Shanghai) optical imaging system. In vivo imaging for detecting ROS NO and RNS using 3D imaging quantitative imaging system (PerkinElmer, USA).

### 2.4 *In vitro* photodynamic assay

The 1,3-Diphenylisobenzofuran (DPBF, 40 μL) solution and 1000 μL of sample solution were added to the 96-well plate and mixed [39]. The laser was irradiated at 1064 nm wavelength with refraction power of 1.0 W/cm^2^. After irradiation, the OD value at 415 nm was measured by microplate every 1min. $\Phi_{\Delta }^{R}$ is the Φ_Δ_ of methylene blue (MB) free in water given as 52.0 %. Using 200 μL sample solution as blank control, the singlet oxygen yield was calculated according to the following formula:

(1) $$\Phi_{\Delta }^{S}=\Phi_{\Delta }^{R}\frac{k^{S}\;I_{aT}^{R}}{k^{R}\;I_{aT}^{S}}$$

(2) $$I_{aT}=I_{0}\left(1-e^{-2.3A}\right)$$

$\Phi_{\Delta }$ represents singlet oxygen yield, Superscript S and R denote sample and reference, respectively. *k* was the value of ln(DOBF0/DPBF), indicating the slope of the plot plotted on the ordinate/detection time as the abscissa, DOBF0/DPBF represents the OD value of the mixed solution before and after illumination respectively; $I_{aT}$ represents the absorbance of sample solution under illumination, *I0* is the absorbance of the blank solution, A represents the OD value of the solution at the laser illumination wavelength.

### 2.5 Structural optimization of PNIR-II

Calculating the density functional theory (DFT) of the electronic structure of PNIR-II using the B3LYP/6-31G* method. HOMO and LUMO energies of this conjugated polymers were estimated with the following equations: HOMO = −4.996570 eV, LUMO = −2.760720 eV. The bandgaps were estimated with their HOMO/LUMO levels (E_g_^cv^ = E_LUMO_ − E_HOMO_). The first triplet state (*T*_*1*_) energy level was 0.9817 eV, and *ΔE*_*S1-T1*_ = 0.3201 eV.

Calculated from the optimizations of excited states with time-dependent density functional theory at the B3LYP/6-311+G (d, p) level in DMSO.

### 2.6 *In vitro* NO release detection

The released NO concentrations of PNIR-II, PSNO and NPs were tested with a NO kit (DAF-FM DA) after added GSH or not added GSH. The NO gas released from the samples can be transformed into nitrite and measured with a fluor spectrophotometer at a 515 nm wavelength (Ex = 488 nm).

### 2.7 GSH consumption of 2-hydroxyethyl disulfide, PNIR-II and PSNO

The standard curves of the reaction products were obtained by the full reaction of different concentrations of GSH with OPA. The GSH solution (10 mM, 100 μL) was reacted with 2-hydroxyethyl disulfide, PNIR-II or PSNO for 0.5, 1.0, 2.0, 4.0 and 8.0 h. OPA was used to test the remaining GSH and then obtained the GSH consumption at each time point.

### 2.8 Reductive-sensitive linker 2,2'-(propane-2,2-diylbis(sulfanediyl)) bis(ethan-1-ol) (S3) reacts with H_2_O_2_

S3 (7.84 mg, 0.04 mmol) and H_2_O_2_ (3.4 mg, 0.10 mmol) were dissolved in 0.6 mL DMSO-d6. The mixture was incubated at 37 °C. At various time points (0.5, 1.0 h, 1.5 h, 2.0 h, 2.5 h, 3.0 h and 4.5 h, respectively), the reaction was monitored by ^1^H NMR. The characteristic peaks were integrated to calculate the degree of reaction.

### 2.9 Degradation of PNIR-II, PSNO and NPs

PNIR-II (1 mg/mL, 2 mL) was added to a 5 mL centrifugation tube containing GSH (10 mM, 2 mL), then the mixture was incubated at 37 °C for 6 h. PSNO (1 mg/mL, 2 mL) was added to a 5 mL centrifugation tube containing H_2_O_2_ (10 mM, 2 mL), then the mixture was incubated at 37 °C for 6 h. GPC test was carried out after the above solution was dialyzed and lyophilized. TEM photography of the degradation process of nanoparticles.

### 2.10 Bacterial cultivation and growth curve

MDRSA were cultured in TSB medium in a shaking incubator (180 rpm) at 37 °C and harvested at the platform stage by centrifugation at 7500 rpm for 3 min. After washing with PBS for three times, the bacteria were resuspended in PBS for further use. The concentration of bacteria was monitored by measuring the optical density at 600 nm (OD600) using SPECTROstar Omega.

100 μL MDRSA suspension (1 × 10^6^ CFU/mL) in PBS, 100 μL different compounds (PNIR-II, PSNO, PSNO+GSH, NPs, NPs+GSH) TSB solution were added sequentially to the 96-well plate. Subsequently, some treatment groups (PNIR-II+L, NPs+L, NPs+GSH+L) were irradiated under 1064 nm laser. At each specific time point, the OD600 value was measured on multifunctional enzyme marker (SPECTROstar Omega, Germany) to plot the growth curve of bacteria.

### 2.11 *In vitro* antibacterial experiments

The bacterial suspension was diluted to a specific concentration (1 × 10^5^ CFU/mL ∼1 × 10^6^ CFU/mL), and treated with different formulations (PBS, PNIR-II, PNIR-II+L, PSNO, PSNO+GSH, NPs, NPs+GSH, NPs+GSH+L) incubating in a shaker (180 rpm) (In terms of NPs 10.0 μg/mL). The groups of PNIR + L and NPs + L were separately irradiated with a 1064 nm laser at a power density of 1.0 W/cm^2^ for 10 min. At each specific time points, 100 μL solution was remove and the OD value was measured to plot the growth curve of bacteria. At 12 h, the solution (100 μL) was spread on LB agar plates after 1000 × dilution. Then the plates were placed in incubator at 37 °C for 18 h, and the bacterial colonies were counted and calculated.

(3) $$\text{Inhibition}\;\text{rate}=\frac{C_{0}-C}{C_{0}}100\%$$

### 2.12 Live/dead staining of bacteria and biofilm

After the MDRSA were treated with the bacterial viability kit (Invitrogen™ LIVE/DEADTM BacLight™, L13152), the bacteria were collected by centrifugation at 7500 rpm for 3 min. After staining the biofilm for 15 min, rinse it repeatedly with PBS three times. The bacteria were photographed using a CLSM.

### 2.13 Morphology of bacteria after treatments

The bacteria of different treatment groups (according to the above experiments) were incubated for 12 h. The bacteria were collected after centrifuging at 7500 rpm for 3 min and then washed thrice with 0.9 % NaCl. Subsequently, the bacteria were fixed with 2.5 % glutaraldehyde for 4 h at 4 °C. After washing with 0.9 % NaCl three times, the bacterial cells were dehydrated through different concentrations of ethanol (30 %–100 %) for 15 min. The samples were dried overnight and photographed by SEM and TEM.

### 2.14 *In vitro* anti-biofilm experiments

The bacterial suspension was diluted to a specific concentration of 1 × 10^6^ CFU/mL using TSB. Subsequently, 1 mL of bacterial suspension was transferred to a confocal dish and cultured in TSB medium in an incubator under agitation (180 rpm) at 37 °C. After 24 h of incubation, the TSB medium was replaced, and the bacterial suspension was further incubated for 24 more hours to allow for the formation of biofilm. The biofilm was then exposed to various treatment materials, including PBS, PNIR-II, PSNO, NPs and co-incubated for 12 h. For some treatment groups, including PNIR-II+L and NPs+L, radiation treatment with a 1064 nm laser was performed. Following the treatment, the biofilm was washed three times with PBS and allowed to dry completely. Next, a 1 % crystalline violet solution was added to the biofilm, and the sample was stained for 30 min. After staining, the biofilm was washed again three times with PBS. Finally, the crystalline violet was dissolved in the biofilm using a 95 % ethanol solution, and the optical density (OD) value was measured at 590 nm using a multifunctional enzyme marker.

### 2.15 *In vitro* cytotoxicity assay

L929 cells were seeded in 96**-**well plates (1 × 10^4^ cells/well) and incubated with RPMI1640 supplemented with 10 % FBS (150 μL) at 37 °C for 24 h. Then, the cells were treated for 8 h with PNIR-II, PSNO and NPs (without laser irradiation) at 10 μg/mL to 50 μg/mL. Thereafter, the cellular viability was assessed via an MTT colorimetric assay. In brief, MTT reagent (10 μL of a 5 mg/mL solution in PBS buffer) was added to each well and the plates were further allowed to incubate with cells for another 4 h. Acidified SDS solution was then added (100 μL/well) and the plates were kept in the dark for an additional 12 h. Measurements of absorbance were subsequently made with a Bio-Rad plate reader (Spectra Max M3) at 570 nm (peak absorbance) and subtracted at 650 nm (background absorbance).

### 2.16 *In vivo* biosafety assessments

The healthy mice were intravenous (*i.v*) injected with PBS, PNIR-II, PSNO, NPs (3 mg/mL; 100 μL) every other day for seven times. At day 14, the serum of ocular blood from mice was obtained to detect the biomarkers (aspartate transaminase (AST), alanine aminotransferase (ALT), alkaline phosphatase (ALP), gamma-glutamyl transferase (GGT), and blood urea nitrogen (BUN). serum albumin (ALB), total protein (TP), creatinine (CRE), total bilirubin (TBIL)) and the major organs including heart, liver, spleen, lung, kidney were separated for hematoxylin-eosin (H&E) staining.

### 2.17 *In vivo* biofilm model

All animals were treated and cared for in accordance with the National Research Council's Guide for the care and use of laboratory animals and under the supervision and assessment by the SPF Animal Department of Clinical Institute in China-Japan Friendship Hospital (Approval no. zryhyy 12-20-08-3).

A wound model was established in mice and left to stabilize for 24 h until no bleeding. The wound was then fixed with sterile tape, followed by an injection of bacterial suspension (1 × 10^7^ CFU/mL, 100 μL). After 24 h, an additional 100 μL of TSB solution was added to promote bacterial growth. The mouse wound site was observed after 12 h, and biofilm formation was determined using cryosection and Gram staining.

Mice at 24 h post-infection was randomly divided into seven treatment groups: PBS, PNIR-II, PNIR-II +L, PSNO, NPs, NPs+808 nm laser and NPs +1064 nm laser (1.0 mg/mL, 200 μL), and NPs were intravenously injected one time. Laser irradiation as above was applied to the PNIR-II +L and NPs + L group after each injection (after 24 h). The infected areas, and the damaged areas, and the body weights were measured. The infected skin tissues after different treatments were collected and fixed in 4 % fixative solution, subjected to H&E staining, and finally examined using a digital microscope. The laser power and radiation time were 1.0 W/cm^2^ and 10 min according to the reported literature [42,43].

## 3 Results and discussions

### 3.1 Preparation and photodynamic performance of polymer

**Fig. 1:**
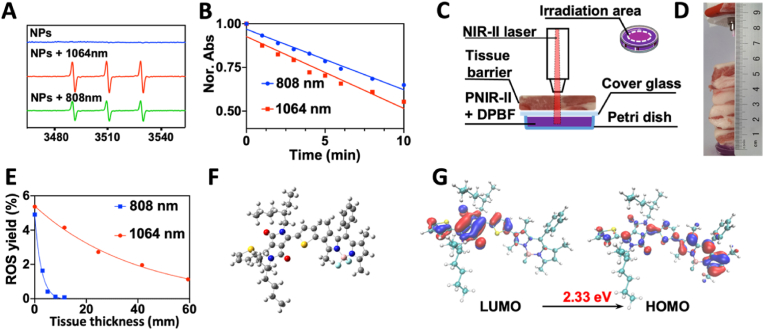
The photodynamic performance of PNIR-II. (A) ESR spectra of PNIR-II (blue) when irradiated by a 1064 nm laser (red, 1.0 W/cm^2^) and an 808 nm laser (green, 1.0 W/cm^2^) for 10 min. (B) Normalized DPBF degradation (monitored at 415 nm) induced by different compounds upon 808 nm (blue) and 1064 nm laser irradiation (red). (C) Schematic representation of deep therapy. (D) Sample setup with a tissue barrier placed on top of a tube containing PNIR-II. (E) Quantitative analysis of ROS produced by NPs (1.0 mg/mL) irradiated with an 808 nm (blue) or a 1064 nm (red) laser under different thick of tissue barrier. (F) Structural optimization and (G) HOMO – LUMO distributions of the PNIR-II repeating unit by DFT calculations.

Scheme S1 presents a comprehensive methodology of synthesising PNIR-II, whereas Scheme S2 displays the approach to synthesise PSNO. ^1^H and ^13^C Nuclear Magnetic Resonance (NMR) analyses verified the accuracy of these chemical structures (Figs. S1–S8). Additionally, GPC data determined that the average molecular weights (*M*_*w*_) of PNIR-II and PSNO were 14.49 kg/mol and 10.18 kg/mol, respectively (Fig. S9), indicating the successful synthesis of the polymers. Next, the UV-spectrum of PNIR-II was detected and two absorption bands in the UV–vis spectrum: 450–810 nm and 989–1091 nm (Fig. S10) were observed. In addition, the extinction coefficient of NPs was ∼33.24 L g^−1^ cm^−1^ at 808 nm, while that at 1064 nm showed a superior extinction coefficient of ∼97.26 L g^−1^ cm^−1^. This indicated that PNIR-II could be excited by 808 nm (NIR-I) and 1064 nm (NIR-II) lasers. Furthermore, the EPR spectra dem a triplet signal (1:1:1) characteristic (a hyperfine coupling constant *A*_*N*_ = 16.28 G and a *g*-value = 2.0055) of a singlet oxygen (^1^O_2_) irradiated by a 1064 nm laser (1.0 W/cm^2^). In contrast, this signal attenuated under 808 nm laser irradiation (Fig. 1A). Since the type of ROS detected was ^1^O_2_, we inferred that the mechanism of ROS generation in PNIR- II was a type II reaction based on literature reports [40]. Therefore, DPBF was used for the quantitative analysis of ^1^O_2_ production by PNIR-II **(**Fig. 1B and Fig. S11) [39]. The ^1^O_2_ yields Φ_Δ_^808nm^ and Φ_Δ_^1064nm^ were calculated to be 4.92 % and 5.38 %, respectively. Notably, both qualitative and quantitative tests indicated that PNIR-II generated more ^1^O_2_ when irradiated by the 1064 nm laser than when irradiated by the 808 nm laser. This implied that PNIR-II is a competitive PSs for centimetre-level PDT, as it has an NIR-II excitation wavelength.

Since NIR-II laser deeply penetrate tissues, they are necessary to evaluate the deep therapy efficiency of the PNIR-II [25,26]. Porcine tissues were used as a scattering medium and a tissue barrier (Fig. 1C, Figs. S12–13). When the distance between the test sample and the light source was kept constant, where PNIR-II was inserted under a 1.15 cm-thick tissue, it produced a large amount of ^1^O_2_ with a Φ_Δ_^1064nm^ of 4.14 % within 10 min of irradiation. However, increasing the thickness of the tissue barrier to 2.46 cm reduced Φ_Δ_^1064nm^ by 2.72 % (Figs. S14–15). Interestingly, increasing the tissue thickness to 4.02 cm and 5.94 cm decreased Φ_Δ_^1064nm^ to 1.95 % and 1.12 %, respectively (Fig. 1D). This revealed that the corresponding tissue thickness for Φ_Δ1/2_^1064nm^ (half of the original yield) of PNIR-II was approximately 2.65 cm. In contrast, a tissue thickness of 1.15 cm reduced the Φ_Δ_^808nm^ of PNIR-II to 0.08 %. In fact, Φ_Δ_^1064nm^ was approximately 52 times greater than Φ_Δ_^808nm^ at the same mass concentration (1 mg/mL) and tissue barrier thickness (1.15 cm). Even when the tissue thickness was reduced to 0.52 cm, Φ_Δ_^808nm^ only increased to 0.41 % (Fig. 1E). Hence, Φ_Δ1/2_^808nm^ corresponded to a tissue thickness of 0.14 cm. Thus, under the centimeter-level tissue barrier, the excellent ^1^O_2_ producing capacity of PNIR-II could be attributed to the better sensitivity of the 1064 nm laser than that of the 808 nm laser. Importantly, the excitation wavelength of PNIR-II was in the NIR-II range.

Furthermore, with the help of density functional theory (DFT), the electronic structure of PNIR-II was calculated using the B3LYP/6-31G* method. Owing to computational power limitations, one repeating unit –(–D–A_1_–D–A_2_–)– of PNIR-II in water was used as the computational model. As shown in Fig. 1F, DPP, thiophene, and BODIPY were found to be in the same plane in the organised PNIR-II electronic structure, although the alkane chain of DPP was significantly twisted. Of note, a certain twist angle was observed between thiophene and BODIPY, which could lower intermolecular contacts and effectively avoid aggregation or π–π stacking (non-radiative attenuation) of the PNIR-II. Additionally, the highest occupied molecular orbital (HOMO) was highly localized on the DPP, whereas the lowest unoccupied molecular orbital (LUMO) was localized on the BODIPY, the distribution of the HOMO–LUMO orbital charge was thoroughly scattered (Fig. 1G), signifying the excitation of twisted intramolecular charge transfer. This transfer enhanced the intersystem crossing efficiency and sensitised the photodynamic effect of PNIR-II [42]. In addition, *T1* energy level (0.9817 eV) of PNIR-II was higher than oxygen sensitization threshold (0.98 eV), which means that PNIR-II could theoretically serve as a photosensitizer. More significantly, PNIR-II exhibited relatively small *ΔE*_*S1*_*-*_*T1*_ (0.3201 eV), which was very favoring high ISC efficiency [30]. The structural rigidity of PNIR-II plays a supportive role in improving the stability of triplet excited states. These two factors together contribute to the better reactive oxygen yield of PNIR-II. The photothermal performance test results showed that PNIR-II did not have photothermal performance (Fig. S16). To our knowledge, PNIR-II was the first NIR-II PSs that had PDT and fluorescence without PTT (Table 1). More importantly, compared with the existing PSs [30], which can be excited by different laser (808 nm and 1064 nm), PNIR-II can generate more ROS under 1064 nm laser radiation. Indeed, PNIR-II was proved to be a NIR–II–triggered PSs that could provide deep therapy.

**Table 1:** Photophysical properties of polymeric NIR-II PS (PNIR-II) and other PSs.

λ_abs_^a^	λ_em_^b^	Electronic structure^c^	Range^d^	Type^e^	Ref
808	1100	A-D-A-D-A	NIR-I	Fl-PDT	[31]
856	1035	A-D-A	NIR-I	Fl-PDT	[32]
668	689	D-A	NIR-I	Fl-PDT	[44]
1019	1070	D-A	NIR-II	Fl-PDT-PTT	[30]
1054	1407	(D-A)_n_	NIR-II	Fl-PDT-PTT	[45]
1043	1137	–(–D–A_1_–D–A_2_–)_n_–	NIR-II	Fl-PDT	This work

a Maximum UV–vis absorption wavelength, in nm.

b Maximum fluorescence emission wavelength, in nm.

c Molecular design strategies.

d The category of excitation light.

e Molecular properties.

### 3.2 Preparation and characterization of NPs

**Fig. 2:**
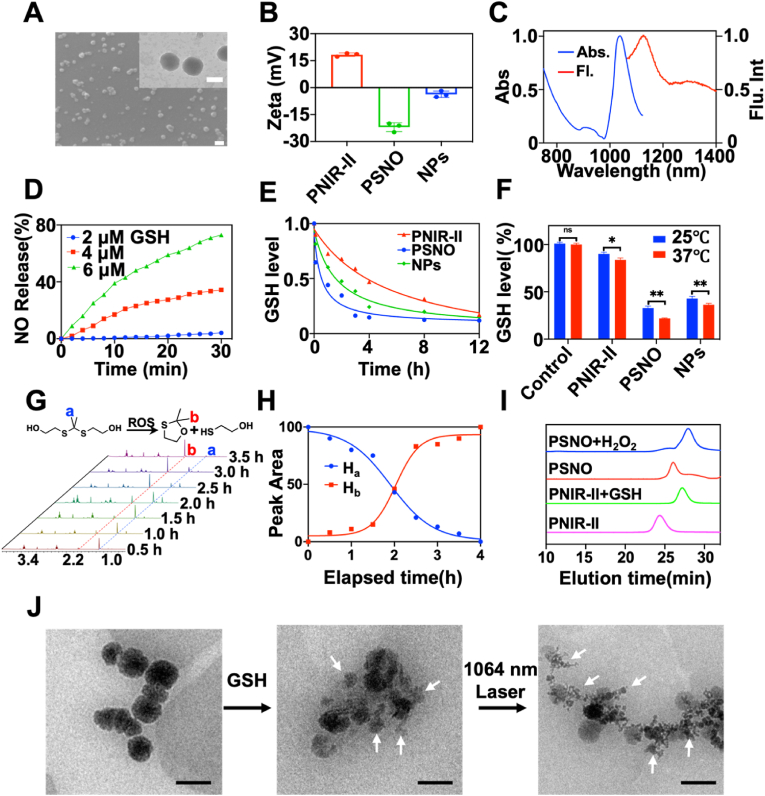
Characterisation and performance analysis of PNIR-II, PSNO, and NPs. (A) SEM image of NPs, the illustration is of transmission electron micrographs images. Scale bar: 100 nm. (B) Zeta potentials of PSNO, PNIR-II, and NPs. (C) Normalized excitation and emission spectra of NPs (1.0 mg/mL, PBS buffer). (D) NO releasing rates of NPs (1.0 mg/mL) stimulating by varying concentrations of GSH. (E) Residual GSH levels consumed by PNIR-II, PSNO and NPs (1.0 mg/mL). (F) GSH consumption levels of various groups within 2 h of irradiation *in vivo* (37 °C) at room temperature (25 °C). (G) Breakdown of S3 by H_2_O_2_ monitored by ^1^H NMR. The proton H_a_ (blue) is the characteristic peak of S3 and H_b_ (red) is the characteristic peak of the S3 degradation product. (H) ^1^H NMR monitoring of the degradation of S3 monomeric compounds by H_2_O_2_ (10 mM), which simulated ROS. Kinetic fitting diagram of the relative peak area ratio of proton H_a_ (blue) and proton H_b_ (red) and the reaction time during S3 degradation. (I) GPC monitoring of polymer degradation triggered by GSH and H_2_O_2_ (10 mM, reaction time was 12 h). (J) TEM image of NPs degradation process. Scale bar: 500 nm. n = 3, *p < 0.05, **p < 0.01, and ***p < 0.001.

For therapeutic applications, we constructed NPs that had a diameter of 115 ± 4 nm (Fig. 2A) and a zeta potential of −3.7 mV (Fig. 2B), by electrostatically assembling them with positive PNIR-II (18.3 mV) and negative PSNO (−22.2 mV). NPs were found to have a spherical morphology and good dimensional stability. Moreover, their particle size measured by DLS (Fig. S17) did not change in 5 days (Fig. S18), thereby verifying their successful construction. Additionally, NPs exhibited an emission peak at 1137 nm (Fig. 2C), and their NIR-II fluorescence intensity gradually increased with increasing concentration, either when excited by the 808 nm or 1064 nm laser (Fig. S19), and the fluorescence quantum yield was calculated to be 2.31 % (Fig. S20). Thus, having an emission wavelength in the NIR-II range enabled NPs to be used as bio-imaging agents. Subsequently, GSH-triggered NO release from the NPs was investigated (Fig. 2D). Remarkably, NPs released a significant percentage (74.3 %) of NO in the presence of 6 μM GSH (to simulate biofilm), whereas they released 41.5 % NO when stimulated with 4 μM GSH. However, in the presence 2 μM GSH (to simulate normal tissue environment), they generated 2.7 % NO, which was compatible with the normal tissues. These results indicated that the release of NO by NPs was sensitive to GSH concentration, thereby favouring the selective eradication of biofilms that typically overexpress GSH.

GSH consumption was further assessed by the NPs using the previously reported the o-phthalaldehyde (OPA) method [46,47]. As shown in Fig. 2E, NPs had a much higher GSH consumption efficiency (72.2 %) than PNIR-II (51.1 %) within 4 h of incubation at 25 °C. In contrast, PSNO consumed 86.1 % GSH under identical conditions. However, since NPs were assembled from both PNIR-II and PSNO, GSH consumption by NPs was slightly higher than that by PNIR-II but slightly lower than that by PSNO, at identical doses. When the incubation time was reduced to 2.0 h, GSH consumption by NPs decreased to 55.4 %. Comparatively, its GSH consumption at 37 °C increased by 1.25 times (Fig. 2F). Therefore, NPs were effective at releasing NO *in vivo* and could thus reverse the hypoxic and GSH overexpressing environment in a biofilm.

The disulphide bonds of PNIR-II and the thioketal bonds of PSNO determine the biodegradability of the NPs [48]. PSNO degradation was evaluated by monitoring the dynamic degradative process of its main chain (2,2'-(propane-2,2-diylbis(sulfanediyl)) bis(ethan-1-ol) (S3, see SI). Interestingly, a distinctive peak of S3 (H_a_) at 1.52 ppm disappeared and a new characteristic peak (H_b_) appeared at 2.07 ppm, suggesting that S3 was entirely damaged within 4 h (Fig. 2G and H). Furthermore, PNIR-II biodegradation was assessed in the presence of GSH (10 mM, mimicking the MDRSA biofilm) based on the *Mw* changes of the polymers. GPC monitoring data revealed that the *Mw* of PNIR-II decreased from 14.49 kg/mol to 5.58 kg/mol, whereas that of PSNO decreased from 10.18 kg/mol to 5.02 kg/mol. These results demonstrated that both the polymers were biodegradable (Fig. 2I). Finally, TEM was employed to monitor the degradation processes of the NPs. Upon exposure to overexpressed GSH, the NPs started to disintegrate while maintaining their spherical shape. Subsequent irradiation with a 1064 nm laser led to the complete disintegration of the NPs, leaving behind small fragments (Fig. 2J). These findings provide further evidence that the NPs can be entirely degraded through a dual response to both GSH and ROS.

### 3.3 *In vitro* antibacterial properties

**Fig. 3:**
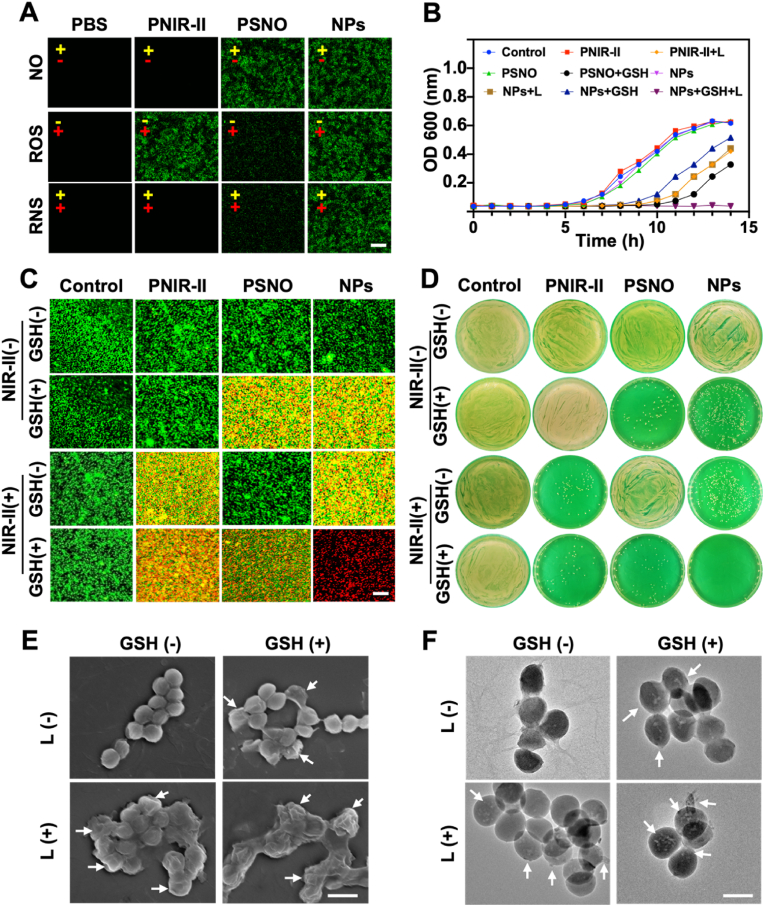
*In vitro* antibacterial effect of PNIR-II, PSNO, and NPs against MDRSA. (A) CLSM images of PNIR-II (5.0 μg/mL), PSNO (5.0 μg/mL), and NPs (5.0 μg/mL) producing NO, ROS, and RNS using probe DAF-FM DA (λ_ex_/λ_em_: 488/515 nm), DCFH-DA (λ_ex_/λ_em_: 488/525 nm), and R21 (λ_ex_/λ_em_: 488/516 nm), respectively. Yellow means GSH; red means NIR-II laser; (+) means with and (−) means without the corresponding conditions. Scale bar: 10.0 μm. (B) Growth curve of MDRSA in different treatment groups. (C) CLSM images of live (greed)/dead (red) staining in different treatment groups; SYTO9, green and Propidium Iodide, red. Scale bar: 5.0 μm. (D) MDRSA colony-forming units in different treatment groups. (E) SEM images of MDRSA in different treatment groups. Scale bar: 1.0 μm. (F) TEM images of MDRSA in different treatment groups. Scale bar: 500.0 nm.

The in vitro antibacterial activity of the NPs was assessed using MDRSA (220024-1B). Remarkably, CLSM (Fig. S21) detected the NPs (red fluorescence) around the MDRSA (green fluorescence), indicating that NPs adhered to the bacterial surface. Scanning electron microscopy (SEM) further confirmed this observation (Fig. S21C). Next, we evaluated the ability of the each materials to release NO, ROS, and RNS on the bacterial surface by using a fluorescence probe assay (Fig. 3A) [49]. Notably, NO production was significantly higher in the PSNO+GSH and NPs+GSH groups than that in the PBS group (control). Indeed, NO generation in the NPs+GSH group was 55.9 % of that in the PSNO+GSH group (Fig. S22). Under 1064 nm laser irradiation, a maximum fluorescence intensity (FI) of ^1^O_2_ was captured in PNIR-II (denoted as PNIR-II+L), while the FI captured by NPs+L was only 51.7 % that of PNIR-II+L. Remarkably, the RNS FI of NPs+GSH+L was as high as 96.4 %. This evidence showed that ROS, NO, and RNS released by each material can be continuously accumulated in MDRSA. Thus, the minimum inhibitory concentration of the NPs+GSH+L group was 8 μg/mL, which was 2–4 times lower than that of the other treatments (Figs. S23–S25).

When all the groups were administered with 10 μg/mL dose, only the NPs+GSH+L group completely inhibited bacterial growth (Fig. 3B). In contrast, the MDRSA growth was normal in the PNIR-II, PSNO, and NPs groups, but was partially inhibited in the PNIR-II+L, NPs+L, NPs+GSH, and PSNO+GSH groups within 8–10 h of administration. RNS generated by NPs+GSH+L group had a stronger bactericidal efficiency. This phenomenon was consistent with relevant reports [50,51]. This bactericidal activity was further validated by performing live/dead staining (Fig. 3C and Figs. S26–S27). The bacterial mortality rates in the PSNO, PSNO+GSH, and PNIR-II+L groups were 0.8 %, 62.4 %, and 47.5 %, respectively. Significantly, the NPs+GSH+L treatment group effectively reduced bacteria from 1 × 10^8^ CFU/mL to 0 CUF/mL, which antibacterial efficiency of up to 99.99 % (Fig. 3D and Fig. S28). SEM (Fig. 3E) revealed that the cell membranes of MDRSA were breached in the laser treatment group (NPs+L) because of ROS production. However, the NPs+GSH groups produced NO that caused cell membrane collapse. The severe deformation of MDRSA membranes were clearly observed post NPs+GSH+L treatment. Furthermore, TEM showed that the bacterial cell membranes treated with ROS group (NPs+L) had obvious damage and holes. Bacteria of NO group (NPs+GSH) showed prominent holes. However, many holes appeared in the bacterial cell membrane of the RNS group (NPs+GSH+L). These findings demonstrate that RNS had a better bactericidal effect than other active substances.

### 3.4 *In vitro* anti-biofilm properties

**Fig. 4:**
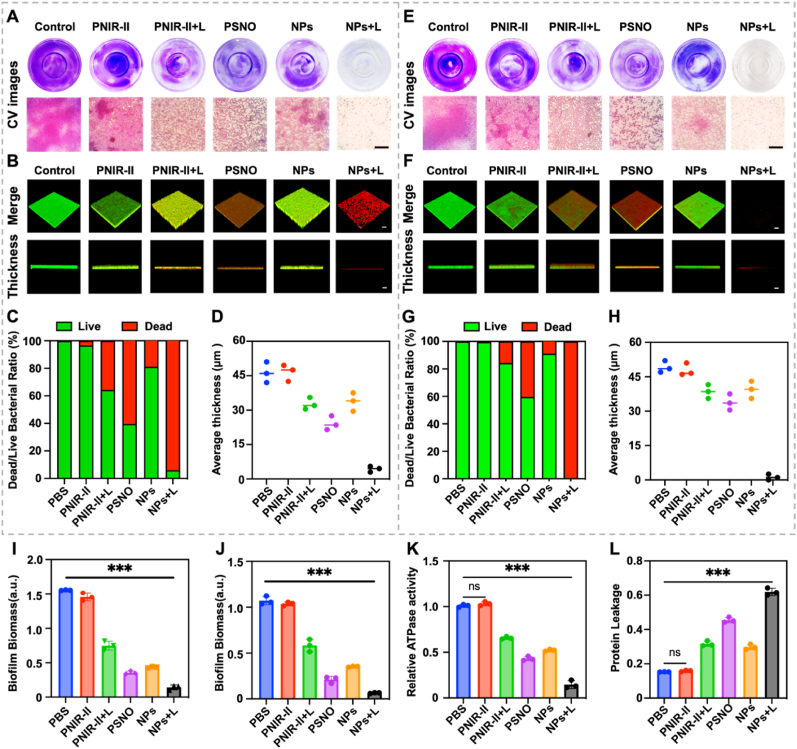
*In vitro* anti-biofilm property by NPs. (A)–(D) The removal effect of different groups on established biofilms. (A) CV images of the removal effect of biofilms formed by different treatment groups. (B) CLSM 3D images of the removal effect of biofilms formed by different treatment groups. (C) Live/death ratio of bacteria in biofilms. (D) Biofilm thickness after treatment with different groups. (E)–(H) Biofilm formed after treating bacteria with different groups. (E) CV images of biofilm formed after treating bacteria with different treatment groups. (F) CLSM 3D images of biofilm formed after treating bacteria with different treatment groups. (G) The live/dead ratio of bacteria in the biofilm formed after treatment. (H) The thickness of biofilm formed after treating bacteria with different groups. (I) Different treatment formulas were incubated with the biofilm biomasses for two days and were subsequently irradiated with NIR-II laser for 10 min. (J) Suspended MDRSA was co-incubated with different groups and cultured with biofilms after 10 min of NIR-II laser irradiation. (K) ATPase activity of MDRSA with different treatment groups. (L) BCA leakage of MDRSA in different treatment groups. n = 3 *p < 0.05, **p < 0.01, and ***p < 0.001.

The limited penetrative abilities of traditional drugs hinder their application in biofilm treatment [52]. Therefore, we monitored the dynamic process of NPs entering the biofilm by CLSM. NPs could not fully penetrate the biofilm constructed by MDRSA in 1 h, they mainly aggregated on the biofilm surface and emitted red fluorescence (Fig. S29). Post 3 h, they fully penetrated the biofilm, as red fluorescence at the bottom of the biofilm could be clearly observed. The NPs with neutral potential can effectively reduce the electrostatic repulsion generated by the negatively charged eDNA in biofilm [53], and the small particle size of NPs can penetrate into biofilm more rapidly [54]. The scavenging effect of NPs on the MDRSA biofilms was further evaluated (Fig. 4A–D). Crystal violet (CV) staining was used to assess the capacity of the NPs to eradicate MDRSA biofilms [55,56]. The NPs+L treatment group could effectively remove most of MDRSA biofilm, according to both macro (CV) and micro (CLSM) results (Fig. 4A). The NPs+L group killed most of the bacterial in the biofilm (Fig. 4B). Interestingly, PSNO group biofilm removal efficiency was better than PNIR-II+L group (Fig. 4C–D and Fig. S30A). The reasons for this phenomenon were (1) bacteria protected from ROS damage by GSH in biofilm, (2) NO itself could be a stepwise destroyer of the biofilm [19]. Meanwhile, the NPs+L treatment group had a significant decrease in biofilm thickness (Fig. 4D). These demonstrates that the developed NPs+L had a high MDRSA biofilm-eradicating efficiency. Subsequently, NPs were also found to be effective in inhibiting biofilm formation, as shown by the biofilm inhibition results (Fig. 4E). CLSM results showed that the NPs+L treatment group could significantly inhibit the formation of biofilm (Fig. 4F–H and Fig. S30B). Only the surface portion bacteria of the biofilm died when biofilm formed in the other treatment groups. Relative statistical of CV staining revealed that PSNO and PNIR-II+L removed 77.2 ± 3.2 % and 52.3 ± 5.2 % of the biofilm mass, respectively. Moreover, NPs+L significantly eliminated 93.2 ± 2.4 % of the biofilm mass (Fig. 4I), suggesting that RNS-generating NPs were more effective in eliminating MDRSA biofilms than other treatments. Notably, increasing the irradiation time from 0 to 10 min efficiently eliminated 95.5 ± 2.7 % of the biofilm mass in the NPs+L group (Fig. S31A). The inhibitory efficiencies of PNIR-II+L and PSNO were 49.5 ± 4.1 % and 83.1 ± 3.3 %, respectively. Furthermore, NPs+L had a significant inhibitory efficiency of 95.3 ± 2.6 %, which was 1.9 times and 1.22 times greater than that of the PNIR-II and PSNO treatments, respectively (Fig. 4J). Indeed, the inhibitory effect of the NPs was amplified when the radiation duration was increased. The inhibition rate of NPs+L on MDRSA biofilm was 96.3 ± 2.3 % after 10 min of irradiation (Fig. S31B). The above results indicate that NPs+L can effectively clear biofilms at low doses and inhibit biofilm regeneration.

Since RNS has a direct impact on the physiological metabolism of MDRSA, the bactericidal mechanism of NPs was investigated by assessing the ATPase activity and the bicinchoninic acid (BCA) leak in the biofilm [[57], [58]]. The ATPase activity of the MDRSA biofilm in the NPs+L group decreased by 85.3 % (approximately 8.5 times that of PBS; Fig. 4K), whereas that in the PSNO and PNIR-II+L groups decreased by 57.6 % and 45.4 %, respectively. Meanwhile, BCA leakage in the NPs+L group was 61.3 % (4.0 times that of PBS) and was the maximum among all the test groups (Fig. 4L). This implied that PNIR-II, PSNO, and NIR-II laser irradiation were indispensable for high bactericidal efficiency. Based on the above data, the neutral potential of NPs allowed them to penetrate the biofilm. After NPs enter the biofilm, it begins to release NO by reacting with overexpressed GSH. NO effectively dispersed the biofilm, making its structure not dense. Subsequently, after NIR-II laser irradiation, the ROS produced by NPs reacts with NO to form RNS, which ultimately leads to bacterial cell death and biofilm degradation. Moreover, RNS generation upon NPs+L treatment directly impacted MDRSA metabolism and destroyed the cell membrane structure. We thus speculate that this is the bactericidal mechanism of RNS produced by NPS+L against MDRSA biofilms.

### 3.5 Biosafety of materials

The biosafety of the NPs was evaluated by MTT assay and intravenous injection of NPs into mice. Based on the MTT results (Fig. S32), the NPs were not cytotoxic. The biochemical blood indicators including aspartate transaminase, alanine aminotransferase, alkaline phosphatase, gamma-glutamyl transferase, blood urea nitrogen, serum albumin, total protein, creatinine, and total bilirubin were also found to support this viewpoint. Remarkably, all indicators in the NPs group were similar to those in the control group, indicating that NPs were not cytotoxic at a single dose of 100 μL (3.0 mg/mL) under the condition of seven-time doses (Fig. S33). Additionally, H&E staining further confirmed that the primary organs (heart, liver, spleen, lung, and kidney) treated with the NPs exhibited normal histomorphology without obvious pathological abnormalities (Fig. S34). Moreover, the body weight of the NPs-treated mice steadily increased over 14 days, demonstrating the biosafety of the NPs (Fig. S35).

### 3.6 *In vivo* imaging

**Fig. 5:**
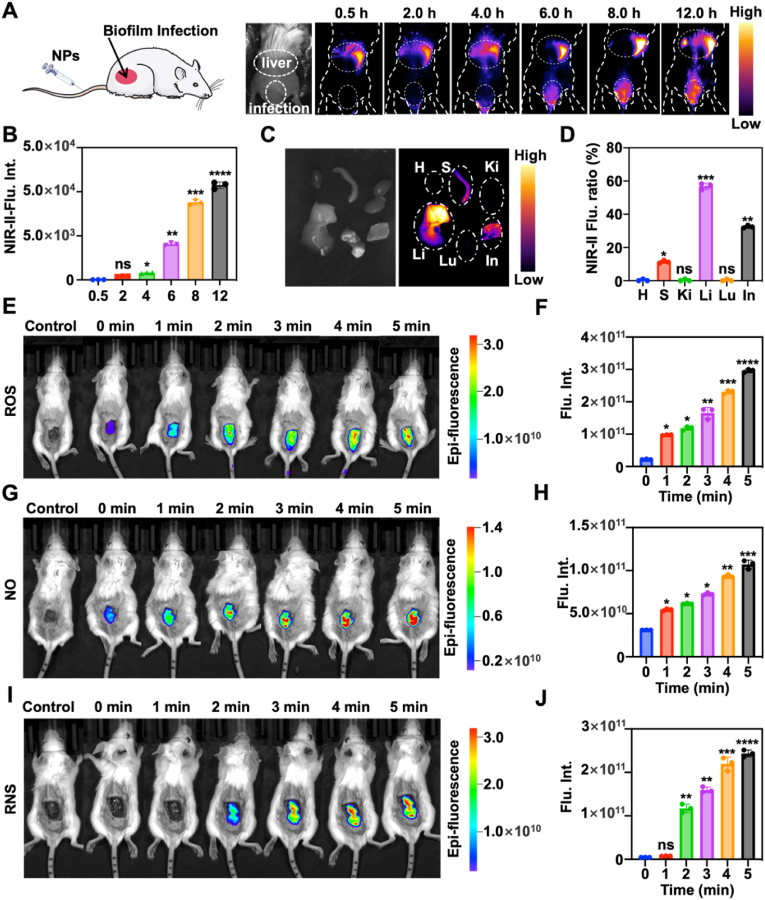
In vivo fluorescence imaging of biofilm infected sites. (A) *In vivo* distribution of NPs via NIR-II bio-imaging. (B) The relative fluorescence intensity of (A) in the infection sites at different times. (C) NIR-II fluorescence ex-vivo imaging of major tissues and organs (S, spleen; H, heart; Lu, lung; Ki, kidney; Li, liver; In, infection areas) after 12 h. Laser parameters: 808 nm, 0.1W/cm^2^. (D) The ratio of NIR-II fluorescence intensity from NPs in various organs and the MDRSA biofilm after 12 h. (E) In vivo imaging for detecting ROS generated by NPs over 5 min by DCFH-DA (λ_ex_/λ_em_: 488/525 nm). (F) Relative fluorescence intersity of (E). (G) In vivo imaging for detecting NO generated by NPs over 5 min by DAF-FM DA (λ_ex_/λ_em_: 488/515 nm). (H) Relative fluorescence intersity of (G). (I) In vivo imaging for detecting ROS generated by NPs over 5 min by R21 (λ_ex_/λ_em_: 488/516 nm). (J) Relative fluorescence intersity of (I). *n* = 3, *p < 0.05, **p < 0.01, ***p < 0.001 and, ****p < 0.0001.

The *in vivo* therapeutic effect of NPs was studied using a classic biofilm mice model of MDRSA, which were wound infection model [59,60]. For this purpose, we isolated MDRSA from clinical MDRSA infection patients who had relapsed after multiple rounds of antibiotic therapy. Firstly, NPs were intravenously injected into the experimental mice, NPs enrichment was studied in the infected area by NIR-II bio-imaging (Fig. 5A). Following this, NPs were enriched at the infection site at 6 h. Moreover, the detected fluorescence intensity increased over time (Fig. 5B). After *in vivo* bioimaging, the biofilm infected area and major organs (including heart, liver, lung, kidneys, and spleen) were visualized *ex vivo* for biodistribution (Fig. 5C and D). This highlighted the *in vivo* ability of NPs to accumulate at the infection site. To avoid the generation of excessive ROS by NPs that cause unnecessary damage to mice, thus laser at 808 nm and 0.1 W/cm^2^ was chosen throughout the whole process for *in vivo* bio-imaging.

Subsequently, bio-imaging was used to further assess the ability of NPs to effectively release ROS, NO and RNS (Fig. 5E–J). There was no fluorescence interference at the biofilm infection site. *In-vivo* bio-imaging of ROS results showed partial fluorescence at 0 min, a phenomenon induced by inflammation-mediated ROS (Fig. 5E). The ROS yield gradually increased with increasing irradiation time of the 1064 nm laser. This evidence that NPs could generate ROS *in vivo* (Fig. 5F). Moreover, after 1 min of laser radiation, the FI of the ROS group was higher than other groups, which indicated that the ROS released by NPs could degrade the thioketal structure of PSNO. The same phenomenon at 0 min, caused by the continuous release of NO from NPs in the infected fraction, was observed in the bio-imaging of NO (Fig. 5G and H). This indicated that overexpression GSH at the biofilm *in vivo* triggered the release of NO. At the same time, this also triggered the disulfide bond degradation of PNIR-II. Interestingly, *in-vivo* bio-imaging results of RNS showed that 1064 nm laser radiation to NPs does not produce RNS in short time (<1 min). It is possible that the amount of ROS generated by laser irradiation of NPs in a short time may not be sufficient for a reaction with NO to form RNS (Fig. 5I). With the increase of 1064 nm laser radiation time, the NPs gradually started to produce RNS (Fig. 5J). This proved that RNS production requires a certain amount of laser radiation time (>2 min). The generation of RNS implied complete degradation of PNIR II and PSNO structures. This also meant complete degradation of NPs.

### 3.7 *In vivo* antibiofilm properties

**Fig. 6:**
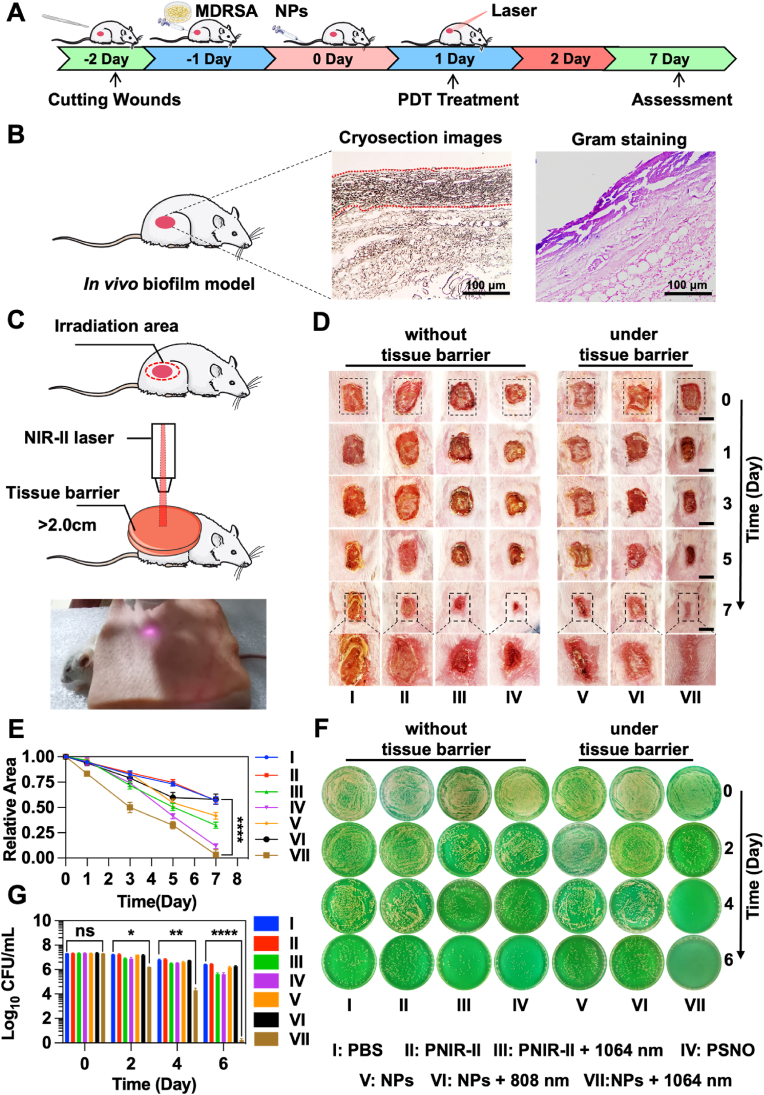
Anti-biofilm effects of different treatment groups against MDRSA (from clinical patients) *in vivo* model. (A) Schematic representation of the development and therapy of the MDRSA biofilm *in vivo* models. (B) Schematic diagram of successful establishment *in vivo* biofilm model, frozen sections, and Gram staining. Scale bar: 100 μm. (C) Schematic diagram of deep treatment *in vivo* biofilm model. (D) Appearance of the MDRSA biofilm infections at different time points. Scale bar: 3 mm. (E) Mean biofilm infection area of mice at different stages of various treatments. (F) Colony count of MDRSA biofilm *in vivo* model at different time points. (G) Relative colony numbers of statistics of MDRSA biofilm *in vivo* model at different time points. *n* = 3, *p < 0.05, **p < 0.01, ***p < 0.001 and, ****p < 0.0001.

The results collectively demonstrate the effectiveness of the RNS group in eradicating bacterial biofilm. To further verify our designing, we established an *in vivo* biofilm model in mice, which we described in detail in Fig. 6A and the experimental methodology [61]. Frozen sections and Gram staining were conducted to confirm that the formation of the *in vivo* biofilm model on mice tissues. The frozen section results showed a dense structure of approximately about ∼90 μm on the normal tissue, which we preliminarily speculated to be an MDRSA biofilm. Subsequently, the Gram staining results revealed that the dense structure was stained purple, a characteristic of Gram-positive bacteria, which further confirmed that the dense structure was MDRSA biofilm tissue (Fig. 6B). To showcase the good penetration ability of NIR-II laser, a 2.2 cm thick tissue barrier was introduced during the treatment process (Fig. 6C and Fig. S36). Therapeutic effects were evaluated on days 0, 1, 3, 5, and 7 for each treatment group (Fig. 6D). Within 0 days of MDRSA transplantation, therapeutic agents (PNIR-II, PSNO, NPs; 200 μL, 1.0 mg/mL) were intravenously administered. On day 0, severe infections were observed in all the groups. On days 1, laser irradiation treatments (808 nm, 1064 nm, 1.0 W/cm^2^) were provided for 10 min under the tissue barrier. The NPs+1064 nm laser group was able to effectively eliminate MDRSA biofilm even under the barrier tissue. The biofilm in the NPs+1064 nm laser group disappeared significantly by day 3, while the other treatment groups still showed severe biofilm presence. By the day 7, wounds treated with NPs+1064 nm laser had successfully healed, leaving scars, whereas other treatment groups still showed obvious biofilms. The relative wound area statistics revealed that although PSNO was able to remove biofilm at the same dose, its clearing efficiency was significantly lower than that of the NPs+1064 nm laser group. Notably, the NPs+808 nm laser group did not exhibit any therapeutic effect under the 2 cm tissue barrier (Fig. 6E). The changes in body weight further confirmed the effectiveness of NPs+1064 nm laser treatment, as mice treated with this method showed recovery and weight gain (Fig. S37). Lastly, plate counting was used to detect bacteria *in vivo* biofilm models at 0, 2, 4, and 6 days. Results showed that while the PNIR-II+L group and the PSNO group were able to inhibit the biofilm, they could not eliminate it. Interestingly, the statistical results indicate that the GSH content in biofilm-infected sites is significantly higher than that in normal tissues (Fig. S38). However, upon the arrival of NPs at the infected site, there was a rapid decrease in GSH content, which can be mainly attributed to the large release of NO from the NPs, and NPs had many GSH-sensitive functional groups. In contrast with other groups, the NPs+1064 nm laser group was able to efficiently kill bacteria within the biofilm even in the presence of a barrier tissue (Fig. 6F). In addition, the bacterial count statistics result showed that on day 2 after treatment, the number of bacteria in the NPs+1064 nm group decreased significantly. On the day 4, the number of bacteria decreased to 10^4^ CFU/mL. Day 6, the bacteria basically disappeared. This indicated that NPs could effectively clear the biofilm *in vivo* (Fig. 6G). These results further demonstrate that the NPs+1064 nm laser can generate RNS and efficiently remove biofilms even under deep tissue barriers.

On day 7, we sacrificed all mice and dissected infected tissues for Gram, H&E, and immunohistochemical (IHC) staining. Blood samples were also collected to analyse inflammatory factors, including proinflammatory cytokines interleukin-6 (IL-6) and tumor necrosis factor (TNF-α), and anti-inflammatory cytokines interleukin (IL-10). Gram staining showed many purple areas in all groups except the NPS+1064 nm laser group, indicating the presence of many Gram-positive bacteria (MDRSA) in the infection site (Fig. S39). H&E results showed a significant decrease in inflammatory cells in the NPs+1064 nm laser group compared to the PBS, PNIR-II, and NPS groups (Fig. S40). At the same time, the obvious skin tissue and layered structure can be observed in the NPS+1064 nm laser group. This demonstrates the effectiveness of the NPS+1064 nm laser group in clearing biofilm and the integrity of tissue recovery. Inflammatory cell statistics show that the PNIR-II+L and PSNO groups also showed a trend of decreasing inflammatory cells. Notably, NPS+808 nm laser had no significant therapeutic advantage compared to the NPs group when barrier tissue was present (Fig. S41). IHC staining showed that the PBS, PNIR-II and NPs groups had higher levels of IL-6, IL-10 and TNF-α expression, indicating inflammation at the infection site (Fig. S42). Simultaneously, the results of simultaneous detection of IL-6, IL-10 and TNF-α in serum showed that, the accumulated levels of IL-6 and TNF-α of NPs+1064 nm group were significantly reduced compared to the PBS group, and interestingly, the accumulated level of IL-10 slightly increased (Fig. S43). In the, Overall, these results demonstrate that single use of laser radiation to produce RNS from NPs can reduce inflammation while clearing biofilm.

## 4 Conclusions

In this study, degradable NPs assembled from polymer PNIR-II and polymer PSNO were designed. Among them, PNIR-II would degrade rapidly when it entered the biofilm microenvironment with overexpressed GSH, due to the GSH-sensitive disulfide structure in the polymer chain. In the meantime, GSH also triggered NO release from PSNO which was degraded when exposed to ROS because of its oxidation-sensitive thioketal structure. PNIR-II could be excited by NIR-II light; even following the passage of the excited light through the 2.6 cm tissue barrier, the PNIR-II maintained 50 % NIR-II PDT efficiency. Particularly, in-depth investigation demonstrated that the –(–D–A_1_–D–A_2_–)_n_– structure composed of two different electron acceptors could enhance the intersystem crossing efficiency, avoid the photothermal effect, and give full play to photodynamic efficiency of PNIR-II. Therefore, after entering the MDRSA biofilm, NPs were triggered to degrade and orderly destroy the biofilm microenvironment, in particularly by accurately releasing NO, and then producing more bactericidal RNS under 1064 nm light irradiation. Hence one can see that the polymer PNIR-II reported here is one of the most efficient NIR-II PSs working under a centimeter-scale tissue barrier. It offers new chances not only for the design of polymeric NIR-II PS, but also for the proof-of-concept application of PDT without PTT in biofilm therapy.

## CRediT authorship contribution statement

**Fanqiang Bu:** Conceptualization, Data curation, Validation, Visualization, Writing – original draft. **Xiaoxu Kang:** Data curation, Formal analysis, Validation. **Dongsheng Tang:** Methodology. **Fang Liu:** Resources, Validation. **Lin Chen:** Methodology, Software. **Pengfei Zhang:** Validation. **Wenli Feng:** Validation. **Yingjie Yu:** Supervision, Writing – review & editing. **Guofeng Li:** Project administration, Supervision, Writing – review & editing. **Haihua Xiao:** Conceptualization, Investigation. **Xing Wang:** Conceptualization, Project administration, Resources, Supervision, Funding acquisition, Writing - review & editing.

## Declaration of competing interest

We have read and understood your journal's policies, and we believe that neither the manuscript nor the study violates any of these. There are no conflicts of interest to declare.
