# Supplementary material for: Enhancing near-infrared II photodynamic therapy with nitric oxide for eradicating multidrug-resistant biofilms in deep tissues
Source: Bioact Mater. 2023 Nov 25;33:341–54. doi: 10.1016/j.bioactmat.2023.11.006 (PMC10724540; doi:10.1016/j.bioactmat.2023.11.006)
Supplement: Multimedia component 1 [file mmc1.zip › Supporting Information BIOMAT 1559/Supporting Information .docx]

**Supplementary Data**

**Enhancing Near-Infrared II Photodynamic Therapy with Nitric Oxide for Eradicating Multidrug-Resistant Biofilms in Deep Tissues**

Fanqiang Bu^a^, Xiaoxu Kang^a^, Dongsheng Tang^b^, Fang Liu^c^, Lin Chen^d^, Pengfei Zhang^a^, Wenli Feng^a^, Yingjie Yu^a^, Guofeng Li^a^, Haihua Xiao^b^, Xing Wang^a,^*

*^a^ State Key Laboratory of Organic-Inorganic Composites; Beijing Laboratory of Biomedical Materials; Beijing University of Chemical Technology, Beijing 100029, P. R. China*

*^b^ Beijing National Laboratory for Molecular Sciences, Key Laboratory of Polymer Physics and Chemistry and CAS Key Laboratories of Organic Solids, Institute of Chemistry, Chinese Academy of Sciences, Beijing, 100190, P. R. China*

*^c^ Department of Oncology of Integrative Chinese and Western Medicine, China-Japan Friendship Hospital, Beijing, 100029, P. R. China.*

*^d^ College of Chemistry and Chemical Engineering, Qiqihar University, Qiqihar 161006, P. R. China*

* Corresponding authors

E-mail address: wangxing@mail.buct.edu.cn

**Experimental Section**

***Synthesis of S1:***

References for the specific synthesis of BODIPY 1 and BODIPY S1.[1] BODIPY 1: BODIPY S1: 1H NMR (400 MHz, CDCl3, δ) 7.52 (d, 2H), 7.25 (d, 3H), 2.65 (s,6H), 1.38(s, 6H).

***Synthesis of S2:***

2-hydroxyethyl disulfide (1.66 g, 10.10 mmol), 5-bromothiophene-2-carboxylic acid (5.22 g, 25.25 mmol), 1-ethyl-3-(3-dimethylaminopropyl) carbodiimide hydrochloride (EDC) (4.84 g, 25.25 mmol), and 4-Dimethylaminopyridine (3.08 g, 25.25 mmol) were dissolved in 100 mL DMF and incubated at room temperature for 24 h. The reaction system was poured into 500 mL water, then extracted with 100 mL ethyl acetate for three times and separated by column chromatography to obtain a white solid S2 with a yield of 71%. 1H NMR (400 MHz, CDCl3, δ) 7.55 (d, 2H), 7.07 (d, 2H), 4.54 (t, 4H), 3.04(s, 4H).

***Synthesis of PNIR-II:***

C1 (850.21 mg, 1.00 mmol), S1 (489.55 mg, 0.85 mmol), S2 (79.77 mg, 0.15 mmol), P(o-tol)3 (23.9 mg, 0.78 mmol), DBa3Pd2 (17.93 mg, 0.019 mmol) were dissolved in 50 mL of degassed toluene, protected by nitrogen gas, and incubated at 120 °C for 5 h, and then the reaction solution was dropped into 500 mL of anhydrous methanol, stood for 30 min, filtered to obtain dark purple precipitation, and finally dried to obtain **PNIR-II**.

***Synthesis of S3:***

Add 25 mL of acetone and 15 mL of thioglycolic acid to a 500 mL volumetric flask, and gradually introduce hydrochloric acid gas (prepared by 100 g NaCl and 100 mL of concentrated sulfuric acid), and then stir at room temperature for 6 h. After the reaction system becomes milky white, the reaction is stopped. Filter and rinse with ethyl acetate/acetone (v/v=1/1). The white solid is 2,2'-(propane-2,2-diylbis(sulfanediyl)) diacetic acid. ^1^H NMR (400 MHz, CDCl_3_, δ) 12.62 (s, 1H), 3.38 (s, 2H), 1.55 (s, 3H).

Weigh 2,2'-(propane-2,2-diylbis(sulfanediyl)) diacetic acid (5 g) and dissolve it in 100 mL anhydrous tetrahydrofuran solution, add LiAlH_4_ (4 g) in batches, and react overnight after heating to 65 °C. After the reaction was stopped, 1 mL NaOH (1 mM) was added, and then anhydrous MgSO_4_ was added. The filtrate was filtered off with suction and separated by column chromatography (MeOH/DCM=1/20) to obtain the monomer **S3**. ^1^H NMR (400 MHz, CDCl_3_, δ) 4.81 (t, 1H), 3.52 (dd, 2H), 2.66 (t, 2H), 1.53(s, 3H).

***Synthesis of PC:***

ROS sensitive linker S3 (2.00 mmol, 392.17 mg), and CHTA (hexahydro-1H, 3H-benzo[1,2-c:4,5-c'] difuran-1,3,5,7-tetraone, 2.11 mmol, 448.63 mg) were suspended in 5 mL anhydrous DMF. After magnetic stirring for 24 h, mPEG5000-OH (2 mmol, 110.03 mg) was added to the reaction mixture. After magnetic stirring for another 24 h, 5 mL mixture was added into 15 mL of deionized water under sonication, followed by dialysis in a dialysis bag (MWCO: 8000 Da). After 72 h, the solution was freeze-dried under reduced pressure to obtained 1012.43 mg light white polymer (PC). The degree of polymerization was analyzed by ^1^H NMR. The molecular weight of polymer was also characterized by GPC.

***Synthesis of PT:***

Dissolve 500 mg of polymer (PC) in 10 mL DMF, add DCC (2.20 mmol, 453.91 mg) and DMAP (2.10 mmol, 256.22 mg). After magnetic stirring for 24 h, then add Bis(2-hydroxyethyl) Disulfide (2.81 mmol, 431.87 mg). After stirring for 12 h, dissolve DTT (8.0 mmol, 1234.01 mg) in 5 mL water and add it to the reaction system and stir for 24 h. Subsequently, followed by dialysis in a dialysis bag (MWCO: 8000 Da). After 72 h, the solution was freeze-dried under reduced pressure to obtained 550.13 mg light white polymer (PT). The degree of polymerization was analyzed by ^1^H NMR.

***Synthesis of PSNO:***

The polymer PC (500 mg) was dissolved in anhydrous 5 mL DMF solution, and added Tert-Butyl nitrite (10 mmol, 1.032 g). After stirring for 24 h kept at 0 °C. Then transfer the dark red reaction system to the ultrafiltration centrifuge tube (5000 Da). Centrifuge for 10 min after adding 5 mL of water (rotating speed 3000 rpm, 0 °C), repeat three times. After 72 h, the solution was freeze-dried under reduced pressure to obtained 457.18 mg pink polymer (PSNO). The degree of polymerization was analyzed by ^1^H NMR. The molecular weight of polymer was also characterized by GPC.

***Preparation process of NPs:***

PNIR- II (10 mg) and PSNO (10 mg) were dissolved in DMF (100 μL) and the solution was placed in ice bath on the magnetic stirrer. Under constant stirring, 900 μL of deionized water was added to above solution. After stirring for 5 min, the NPs were assembled in the solution. It was then centrifuged (Centriprep YM-10®, 10 kDa) and washed tenth with deionized water to remove DMF solution. The concentrated NPs solution was quantified by UV-spectrophotometer. The temperature was kept at 0°C throughout the preparation process.

***Fluorescence Quantum Yield (PLQY) Calculation***

PLQY of the fluorophores was measured in a similar way to literature. The fluorescence spectra in the region of 900-1500 nm were measured b under an 808 nm laser excitation. The fluorescence quantum yield of IR-26 (0.5%) measured in dichloroethane species was used as a reference value [2]. Five different concentrations of PNIR-II were measured, and the linear relationship between the absorbance and fluorescence emission of PNIR-II was plotted. Comparison of the slopes led to the determination of the quantum yield of PNIR-II. The quantum yield was calculated in the following manner:

$\mathrm{PLQY}_{\mathrm{sample}}= \mathrm{PLQY}_{\mathrm{ref}}\frac{{s\mathrm{lope}}_{\mathrm{sample}}}{\mathrm{slope}_{\mathrm{ref}}}(\frac{n_{\mathrm{sample}}}{n_{\mathrm{ref}}})$^2^

n is the refractive index of the solvent.

**Scheme S1.** Synthetic routes to **PNIR-II**.

**Scheme S2.** Synthetic routes to **PSNO**.

**Fig. S1.** ^1^H NMR spectra of **S1** in CDCl_3_.


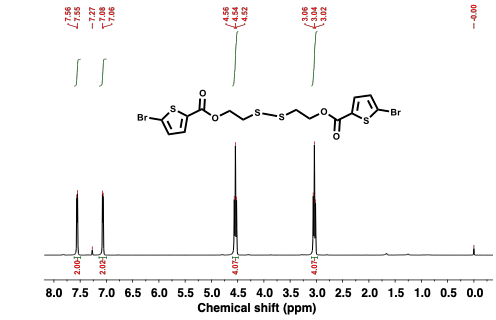


**Fig. S2.** ^1^H NMR spectra of **S2** in CDCl_3_.

**Fig. S3.** ^1^H NMR spectra of **PNIR-II** in CDCl_3_.

**Fig. S4.** ^1^H NMR spectra of pro**S3** in d6-DMSO.

**Fig. S5.** ^1^H NMR spectra of **S3** in d6-DMSO.

**Fig. S6.** ^1^H NMR spectra of **PC** in d6-DMSO.

**Fig. S7.** ^1^H NMR spectra of **PT** in d6-DMSO.

**Fig. S8.** ^1^H NMR spectra of **PSNO** in d6-DMSO.


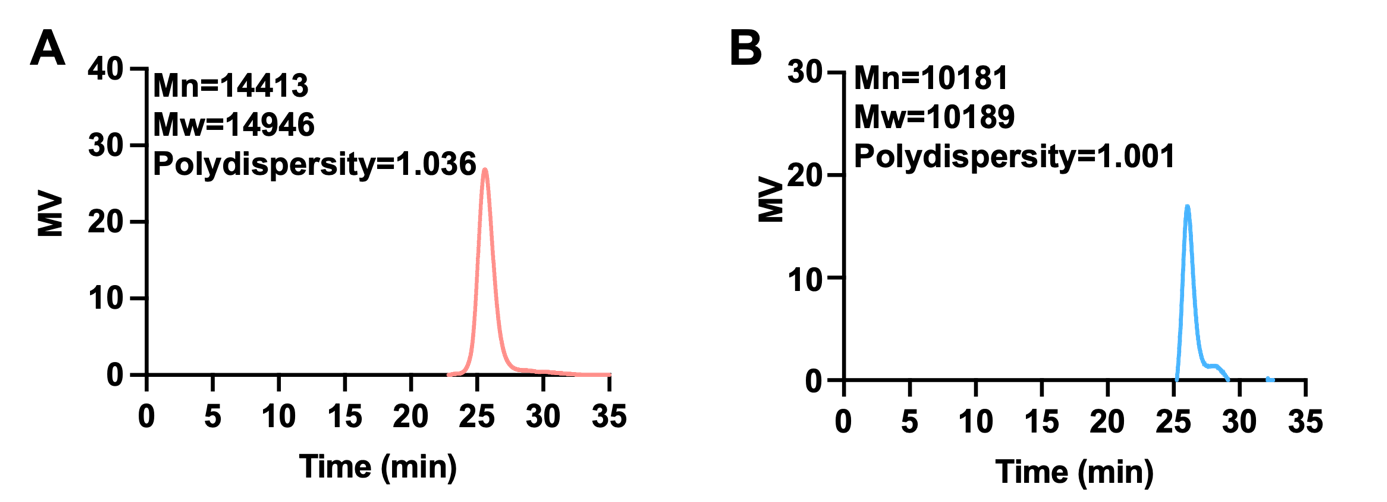


**Fig. S9.** GPC spectra of (A) **PNIR-II** and (B) **PSNO** in DMF.

**Fig. S10.** Normalized UV-excitation spectrum of PNIR-II (20 μg mL^–1^) in PBS buffer.

**Fig. S11.** PNIR-II produce ROS effect by DPBF under (A) 808 nm and (B) 1064 nm laser irradiation.


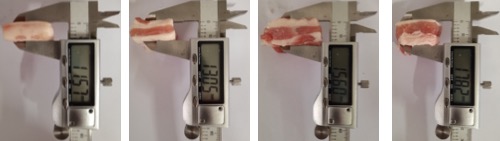


**Fig. S12.** Actual thickness of Meat that simulates human tissue.

**Fig. 13.** Power density of 808 nm and 1064 nm lasers after penetration through tissues with different thicknesses


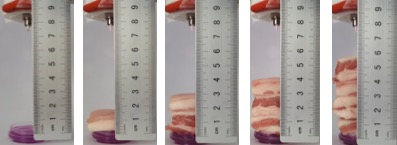


**Fig. S14.** The rate of ROS produced by PNIR-II (1 mg/mL) under NIR-II (1064 nm) laser radiation of tissues with different thicknesses.

**Fig. S15.** The rate of ROS produced by PNIR-II (1 mg/mL) under NIR-II (1064 nm) laser radiation of tissues with different thicknesses.

**Fig. S16.** Temperature variation curves of PNIR-II (1 mg/mL) solutions and water after irradiation by a 1.0 W/cm^2^ 1064 nm laser for 5 min.


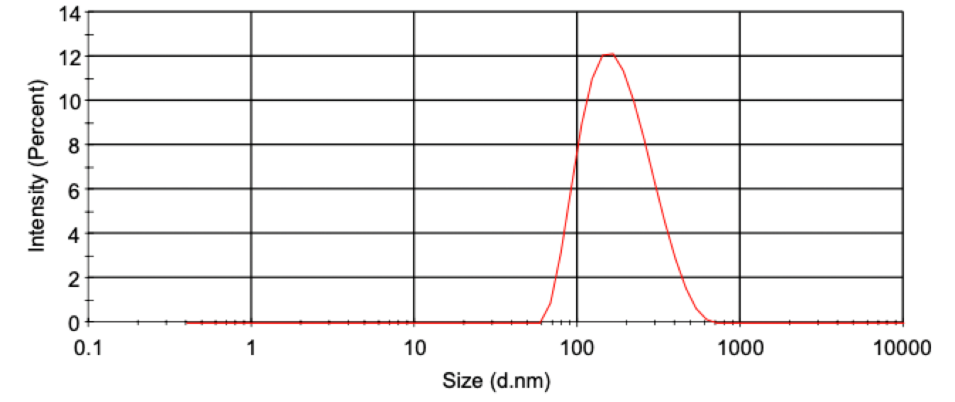


**Fig. S17.** Size distribution of **NPs** by Dynamic Light Scattering (DLS) in PBS. Z-Average (d.nm): 129.3; PDI: 0.139; Intercept:0.949.

*
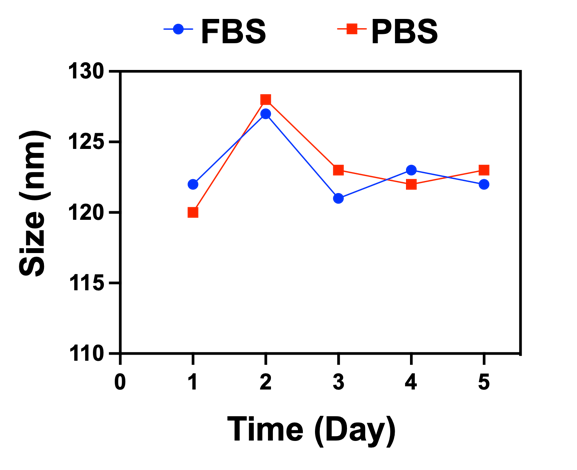
*

**Fig. S18.** The size distribution of **NPs** by DLS within 5 days in PBS (bule) and FBS (pink).


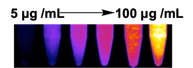


**Fig. S19.** Comparison of **NIR-II** signals of different concentration **NPs** under an 808 nm excitation laser (0.1 W/cm^2^, under 1150 nm filters, exposure time: 200 ms).


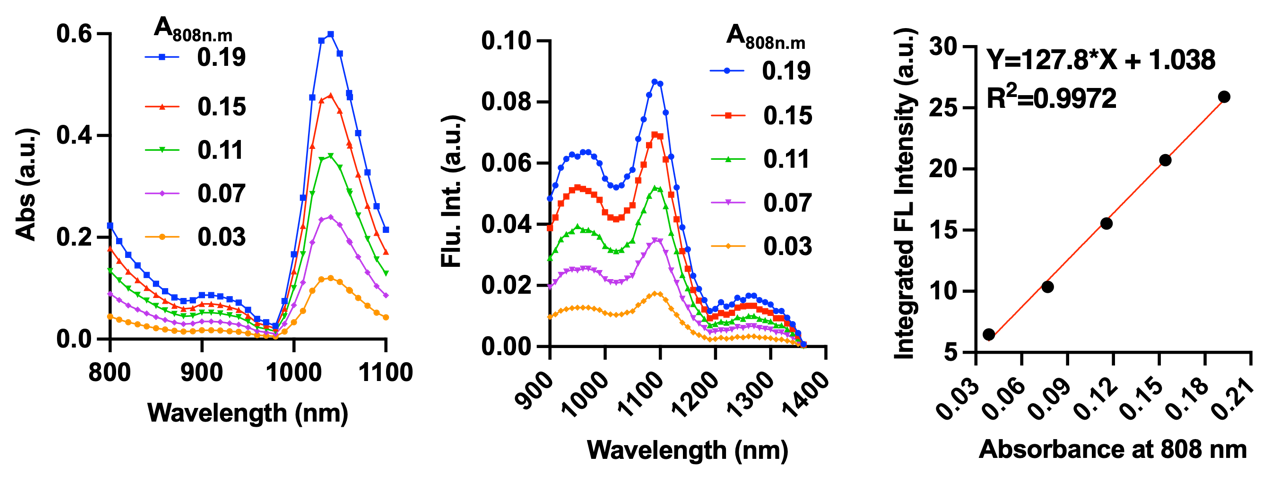


**Fig. S20.** Data in NIR-II fluorescence quantum yield calculation of PNIR-II. UV-Vis-NIR absorption spectra, FL spectra, and a plot of integrated NIR-II fluorescence.

**Fig. S21.** (A) CLSM images of **NPs** and MDRSA were incubated together for 1 h. The red color from **NPs**. The green from SYTO9. Scale bar: 1.0 μm. (B) CLSM 3D image of **NPs** and MDRSA were incubated together for 1 h. Scale bar: 5.0 μm. (C) SEM images of incubate together between **NPs** and MDRSA, Scale bar: 100 nm.

**Fig. S22.** Relative fluorescence intensity of each material produce NO, ROS, and RNS by probe DAF-FM DA, DCFH-DA, and R21.

**Fig. S23.** The MIC of **PNIR-II+L (ROS)** group.

**Fig. S24.** The MIC of **PSNO+GSH** (NO) group.

**Fig. S25.** The MIC of **NPs+GSH+L (RNS)** group.


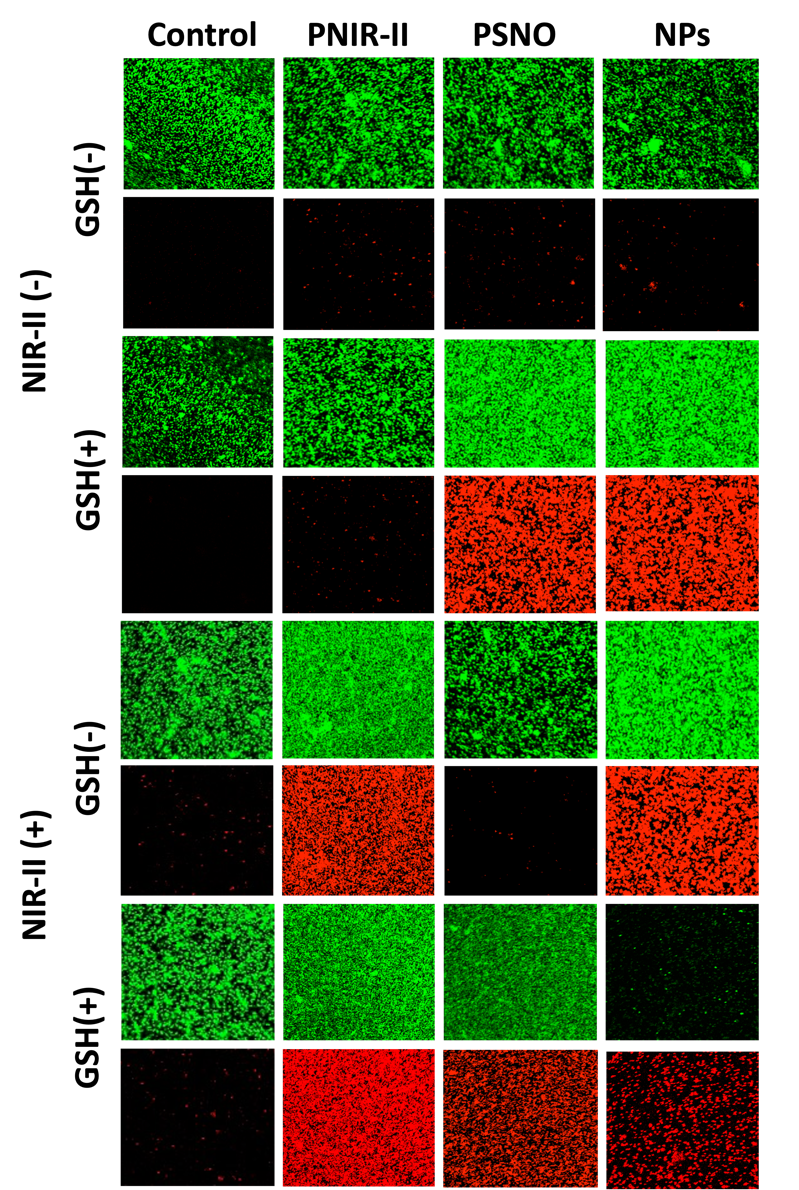


**Fig. S26.** The original data of live/ dead staining assay of each material.

**Fig. S27.** Relative Statistics of Bacterial Fluorescence (Red) of Death in different groups.

**Fig. S28.** Relative colony numbers statistics of Fig. 3D.


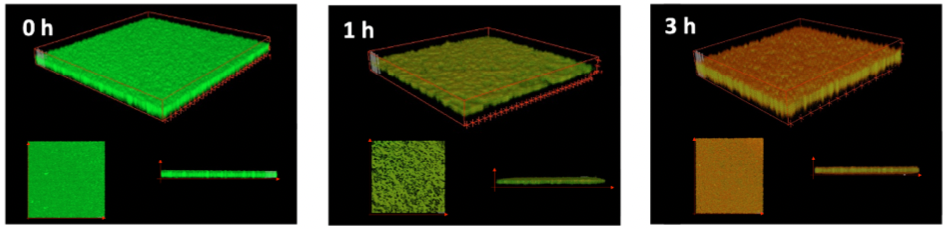


**Fig. S29.** CLSM 3D images of biofilm at 0, 1 and 3 h following NPs penetration. Green, SYTO9-stained live bacteria; Red, Nile red-stained NPs.


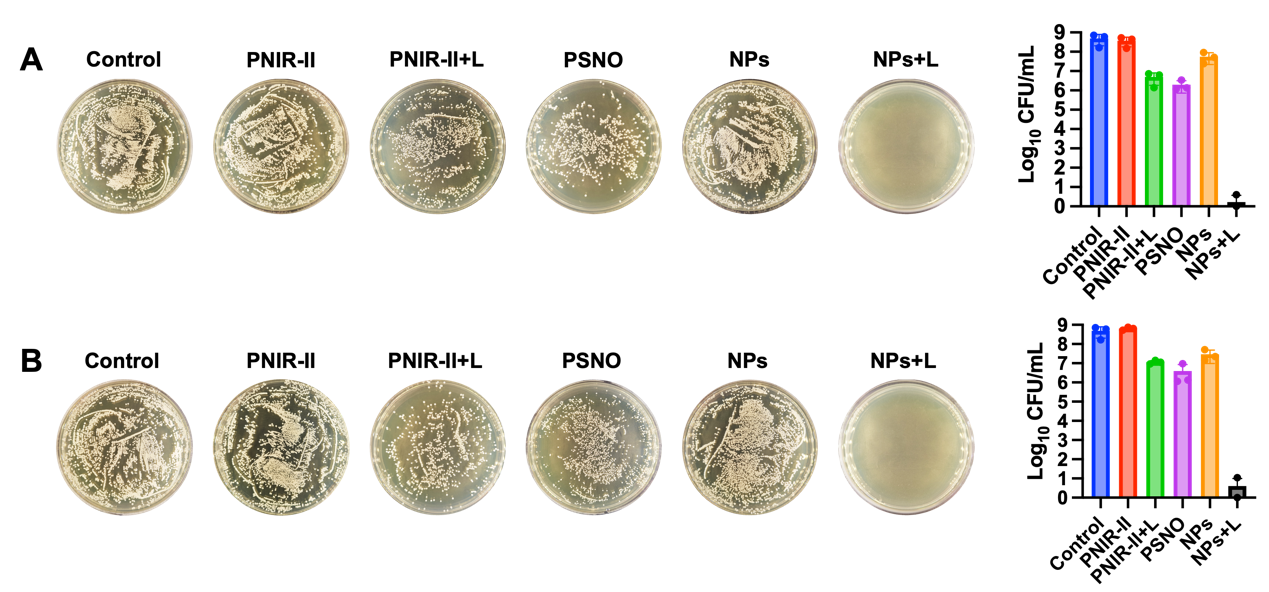


**Fig. S30.** (A) Bacterial statistics of the removal effect of different groups on established biofilms. (B) Bacterial statistics of Biofilm formed after treating bacteria with different groups.


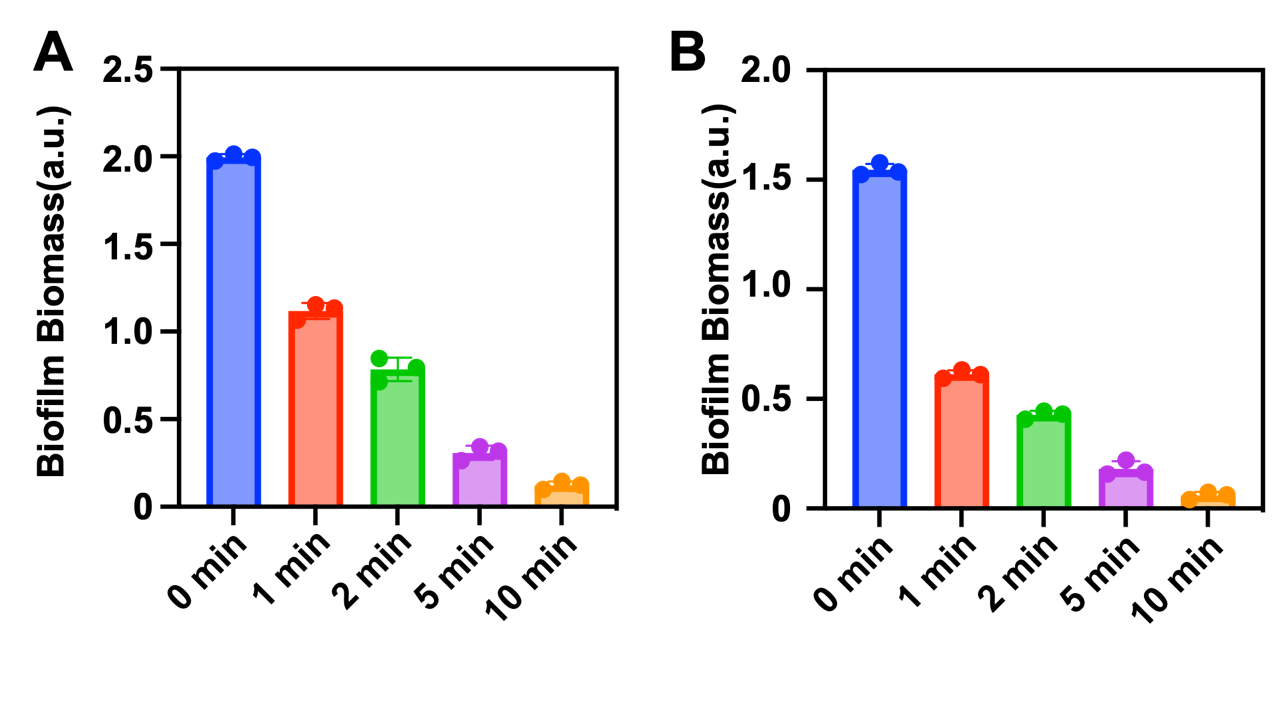


**Fig. S31.** (A) Post incubation NPs were added and then irradiated with NIR-II for different time durations. (B) Suspended MDRSA was co-incubated with NPs and irradiated with NIR-II laser for varying time durations.

**Fig. S32.** Cytotoxicity of **NPs** to human L929 cells *in vitro*.

**Fig. S33.** The biochemical blood indicators of **NPs** by tail vein injection.


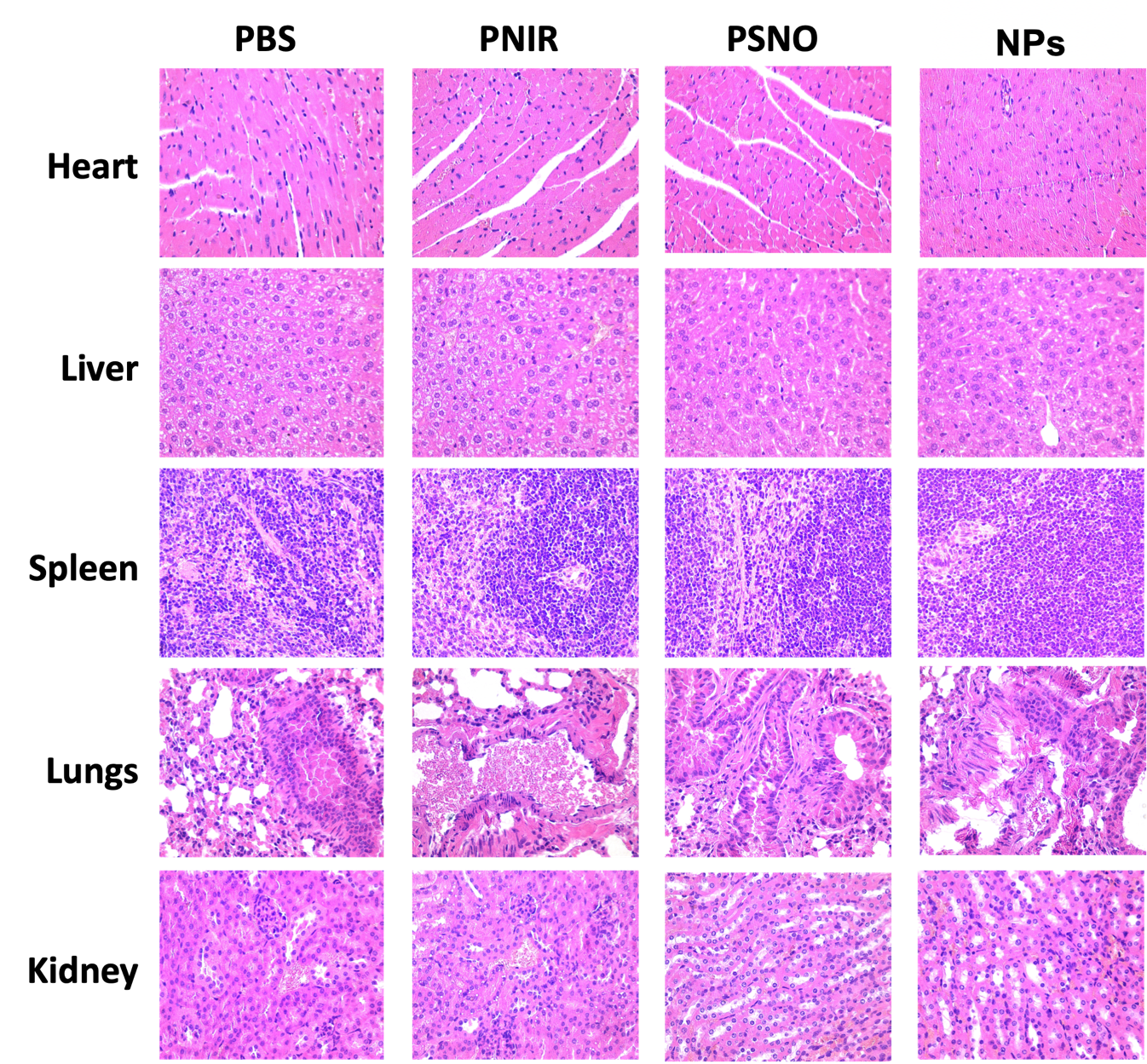


**Fig. S34.** H&E staining of organs in different treatment groups after 14 days by tail vein injection.

**Fig. S35.** The biochemical blood indicators of **NPs, PNIR-II** and **PSNO** by tail vein injection.


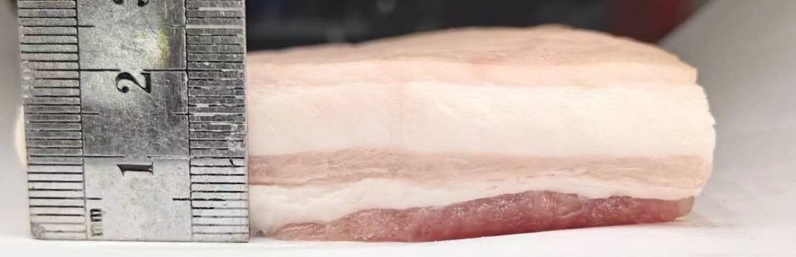


**Fig. S36.** Actual thickness of Meat that simulates human tissue in vivo biofilm model.

**
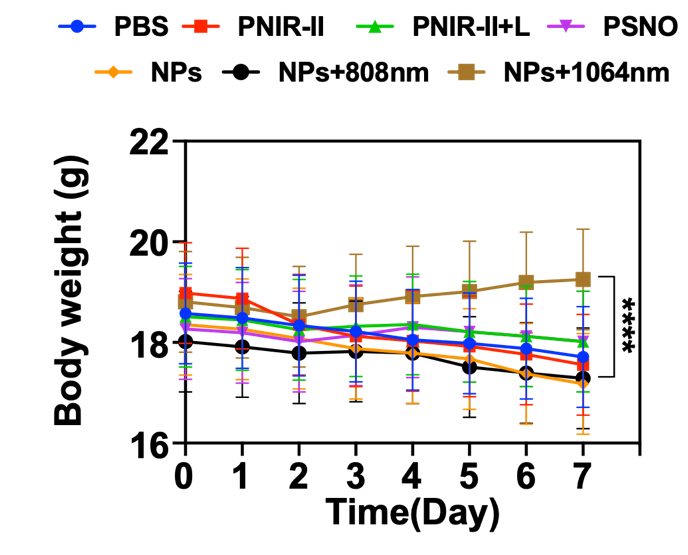
**

**Fig. S37.** Mean body weights of the mice subjected to different treatments.

**Fig. S38.** Residual GSH levels consumed by NPs in vivo biofilm model.


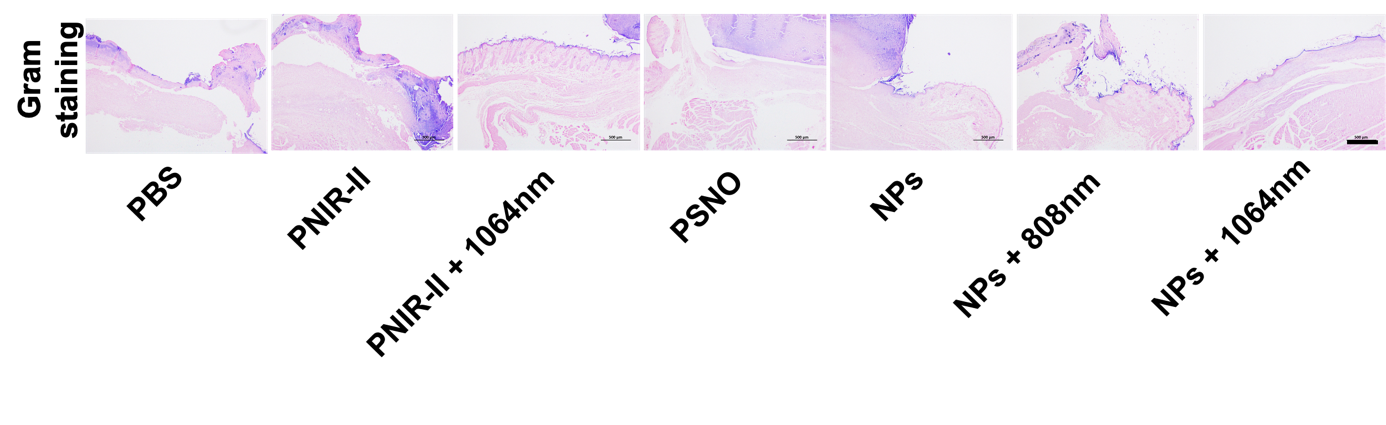


**Fig. S39.** Gram staining of MDRSA biofilm infected area with each treatment groups. Scale bar: 500.0 μm.


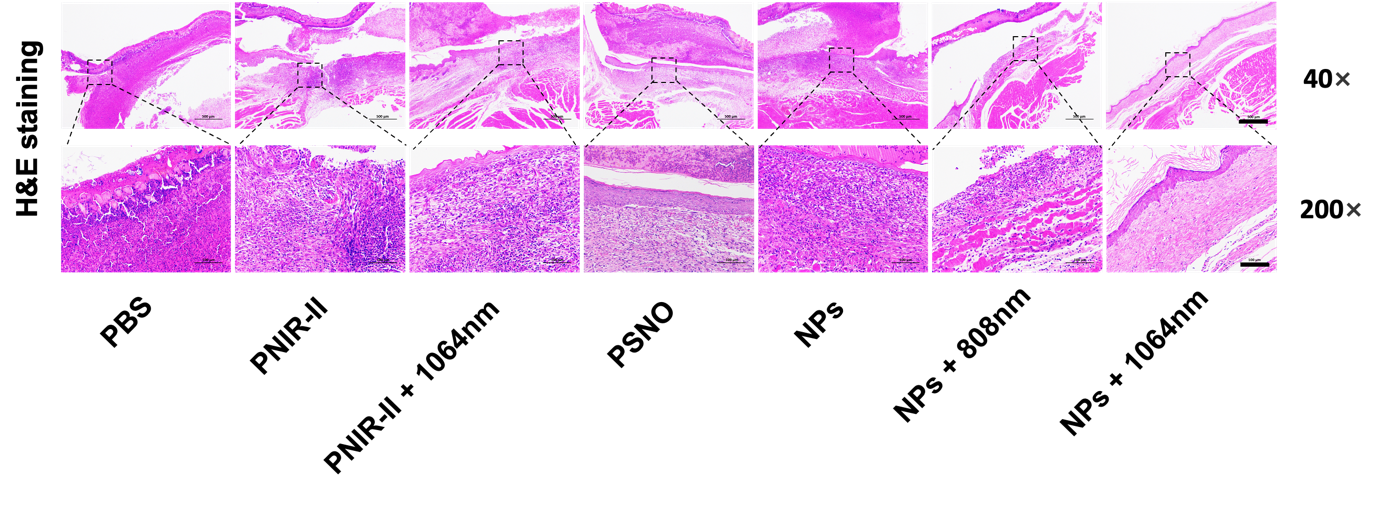


**Fig. S40.** H&E staining of MDRSA biofilm infected area with each treatment groups. Scale bar: 40×, 500.0 μm; 200×, 100.0 μm.

**Fig. S41.** Relative statistics of inflammatory cells at infection sites between different groups.


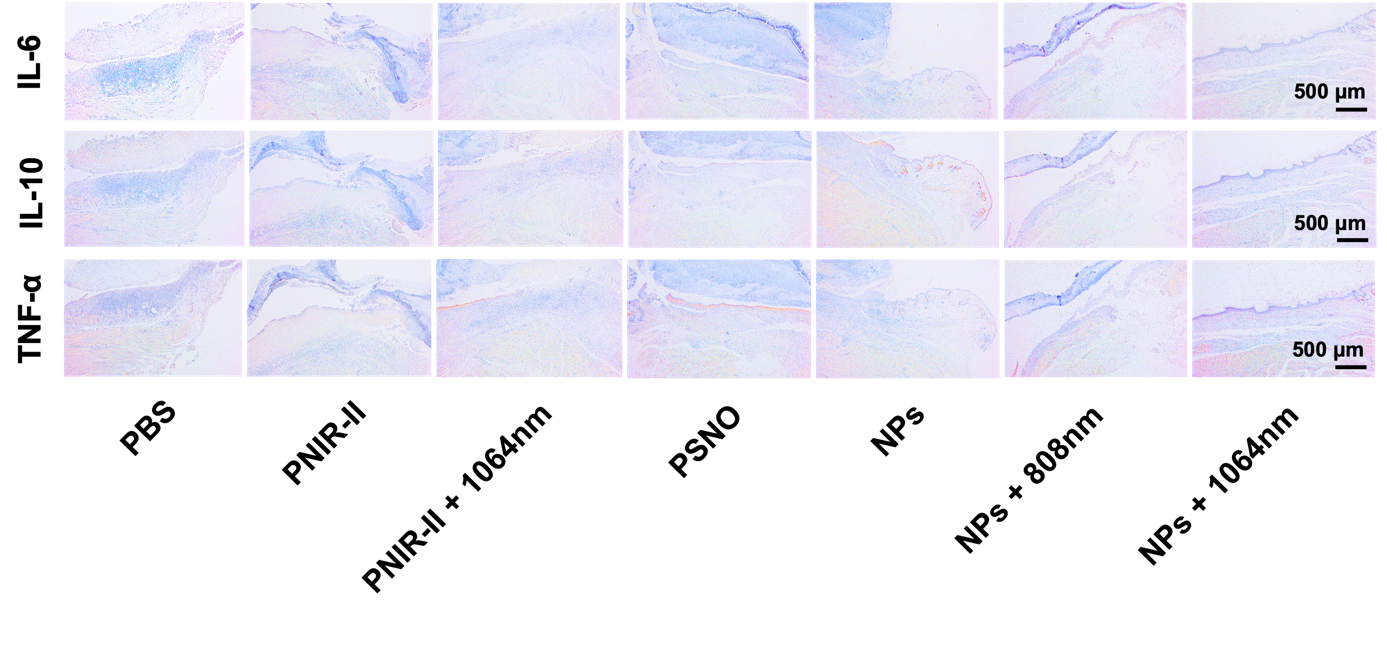


**Fig. S42.** IHC staining of MDRSA biofilm infected area with each treatment groups. Scale bar: 500.0 μm.

**Fig. S43.** Relative statistics of inflammatory factors in serum between different groups.

References.

[1] H. He, S. Ji, Y. He, A. Zhu, Y. Zou, Y. Deng, H. Ke, H. Yang, Y. Zhao, Z. Guo, H. Chen,

Adv. Mater. 2017, 29, 1606690.

[2] Y. Liu, M. Gu, Q. Ding, Z. Zhang, W. Gong, Y. Yuan, X. Miao, H. Ma, X. Hong, W. Hu, Y. Xiao, Angew. Chem. Int. Ed. 2023, 62, e202214875.
